# Circulating large extracellular vesicles as diagnostic biomarkers of indeterminate thyroid nodules: multi-platform omics analysis

**DOI:** 10.1093/bjsopen/zrae139

**Published:** 2024-12-30

**Authors:** Nada M Ahmed, Mohammad M R Eddama, Kevin Beatson, Rijan Gurung, Jigisha Patel, Georges Iskandar, Alaa Abdel-Salam, Abdullah Al-Omar, Richard Cohen, Tarek Abdel-Aziz, Lucie Clapp

**Affiliations:** Institute of Cardiovascular Sciences, University College London, London, UK; Pathology Department, Alexandria University, Alexandria, Egypt; Department of Surgical Biotechnology, Division of Surgery and Interventional Science, University College London, London, UK; Department of Surgical Biotechnology, Division of Surgery and Interventional Science, University College London, London, UK; Department of Surgical Biotechnology, Division of Surgery and Interventional Science, University College London, London, UK; Department of Surgical Biotechnology, Division of Surgery and Interventional Science, University College London, London, UK; Department of Anaesthesia and Perioperative Medicine, University College London Hospitals, London, UK; Endocrine Surgery Unit, University College London Hospitals, London, UK; Endocrine Surgery Unit, University College London Hospitals, London, UK; Department of Surgical Biotechnology, Division of Surgery and Interventional Science, University College London, London, UK; Endocrine Surgery Unit, University College London Hospitals, London, UK; Institute of Cardiovascular Sciences, University College London, London, UK

## Abstract

**Background:**

While most thyroid nodules are benign, 7–15% are malignant. Patients with indeterminate thyroid nodules (specifically Bethesda IV/Thy3f) often undergo diagnostic hemithyroidectomy to reach a diagnosis on final histology. The aim of this study was to assess the feasibility of circulating large extracellular vesicles as diagnostic biomarkers in patients presenting with Thy3f thyroid nodules.

**Methods:**

This was a two-gate diagnostic accuracy study; patients with Thy3f thyroid nodules were age, sex and body mass index matched to healthy individuals. Final histology confirmed benign and malignant diagnoses. Plasma large extracellular vesicle counts were quantified using flow cytometry. Large extracellular vesicle microRNA and protein profiles were identified using next generation sequencing and mass spectrometry, respectively.

**Results:**

A total of 42 patients with Thy3f nodules (22 with cancer, 20 with non-cancer diagnosis) and 16 healthy controls were included. Total large extracellular vesicle concentrations and the concentrations of extracellular vesicles expressing epithelial cell adhesion molecule and the cancer markers atypical chemokine receptor type 7, extracellular matrix metalloproteinase inducer and syndecan-4 were significantly higher in patients with Thy3f nodules (cancer and non-cancer) compared with healthy individuals. In patients with cancerous *versus* non-cancer Thy3f nodules, one microRNA was upregulated: mir-195–3p (*P* < 0.001). Five were downregulated: mir-3176 (*P* < 0.001), mir-205-5p (*P* < 0.001), novel-hsa-mir-208-3p (*P* < 0.001), mir-3529-3p (*P* = 0.01) and let-7i-3p (*P* = 0.02). Furthermore, three large extracellular vesicle proteins (kallikrein-related peptidase11 (KLK11) (*P* = 0.001), *alpha*-1-acid glycoprotein 2 (A1AG2) (*P* <0.001) and small integral membrane protein 1 (SMIM1) (*P* = 0.04)) were significantly upregulated, while 20 large extracellular vesicle proteins were significantly downregulated (most downregulated: chemokine (C-X-C motif) ligand 7 (CXCL7), tubulin beta chain 1 (TBB1), binding immunoglobulin protein (BIP) and actinin alpha 1 (ACTN1) (*P* < 0.001)) in cancerous compared with non-cancer Thy3f nodules.

**Conclusion:**

Circulating large extracellular vesicle miRNA and protein profiles have a high diagnostic value to discriminate between benign and malignant nodules for patients with Thy3f cytology. Further validation for clinical performance will be needed.

## Teaser

This is a proof-of-concept study which demonstrates for the first time the potential of circulating extracellular vesicles to discriminate between non-cancer and cancerous indeterminate thyroid nodules. Mir-195-3p was upregulated, while five other microRNAs were downregulated (mir-3176, mir-205-5p, mir-3529-3p, let-7i-3p and novel-hsa-mir-208-3p) in patients with cancer compared to non-cancerous nodules. Furthermore, three large extracellular vesicle proteins were significantly upregulated (kallikrein-related peptidase11, *alpha*-1-acid glycoprotein 2 and small integral membrane protein 1), while 20 proteins were significantly downregulated. The top two downregulated proteins were chemokine (C-X-C motif) ligand 7 and tubulin β-1 chain. These findings pave the way to a new non-invasive test that could identify patients with malignant thyroid nodules without diagnostic surgery, potentially saving cost, healthcare outcomes and quality of life.

## Introduction

Thyroid cancer incidence has increased three-fold over the past four decades and now has the seventh highest cancer incidence globally. Thyroid nodules are largely benign, however, 7–15% will be malignant depending on factors such as age, sex, radiation exposure and family history^1^. Clinical assessment, imaging and cytology can establish a diagnosis in approximately 80% of nodules^2^; the remaining 20% are categorized as ‘indeterminate’^3^. Specifically, the category of Thy3f in the Royal College of Pathology system^4^ (known as Bethesda IV in the American system^2^) carries 15–30% risk of malignancy^2,5^. Currently, patients with a cytological diagnosis of Thy3f are offered diagnostic hemithyroidectomy to confirm histological diagnosis. Hemithyroidectomy carries risks of metabolic and anatomic surgical complications and the potential need for lifelong thyroid hormone supplementation, impacting patient productivity and quality of life^6^. There is an urgent need for a non-invasive diagnostic test to distinguish malignant from benign Thy3f nodules without the need for diagnostic surgery.

There are currently molecular tests designed to stratify risk on cytology. These include ThyroSeq version 3, Genomic Classifier, Afirma Gene Sequencing Classifier and Xpression Atlas, and combined ThyGeNEXT and ThyraMIR^1^. Despite advances, clinical applicability is hindered by cost, low predictive value, the invasive nature of cytological sampling and lack of Food and Drug Association (FDA) and National Institute for Health and Care Excellence (NICE) approvals^5,7^.

Liquid biopsy is a non-invasive method involving the analysis of molecular alterations of cancer from tumour-derived components in body fluids, such as blood^8^. One blood component showing great promise as biomarkers are extracellular vesicles (EVs). These are anucleate nanosized vesicles bound by a phospholipid bilayer membrane. EVs are shed from body cells, including tumour cells, into the extracellular space and subsequently into blood. This shedding increases when cells undergo proliferation, cell division or apoptosis^9^.

Microvesicles or large EVs (L-EVs, 100–1000 nm) are a subset of EVs that bud directly from the cell membrane and play a role in intercellular communication^10^. They express membrane receptors and contain bioactive cargo including RNA, DNA and proteins that originate from their parent cells^11^. There are advantages of EVs over other blood biomarkers. First, EVs are typically produced in greater quantities compared with other biomarkers like circulating tumour cells and circulating DNA. This is especially important in cancers producing low quantities of these other markers, resulting in low detection rates, as is the case in differentiated thyroid cancers^12^. Second, EV biomarkers are stable as their vesicular membrane protects their contents^13^. For example, EV-enclosed RNAs are shielded from blood-derived RNAses, preventing their degradation^14^.

To this end, transcriptomics and proteomics to characterize EV biomarkers are novel high-throughput approaches to quantify RNA and protein composition. These accelerate biomarker discovery and are gaining traction for biomarker development for many diseases, including cancer^15^.

The aims of this study were to assess circulating L-EV counts through a non-invasive blood test, analyse their membrane surface markers and utilize multi-platform omics to identify potential diagnostic L-EV biomarkers capable of distinguishing between benign and malignant Thy3f nodules.

## Methods

### Patient recruitment and ethical approval

This study adopted a mixed design including a two-gate diagnostic case-control design, using healthy control participants (HCs) and a single-gate classic design for patients with Thy3f nodules. Patients with Thy3f nodules underwent hemithyroidectomy at University College London Hospital between October 2020 and October 2023. HCs were age, sex and body mass index matched and recruited from staff members at University College London. HCs were not on regular medications, not known to suffer from any health conditions and had no symptoms of thyroid disease. Excluded were: children below the age of 18 years, patients with other active cancer or inflammatory conditions and those with acute illnesses. Patient demographics were collected using the hospital electronic health record system (EPIC: 2020 EPIC system Corporation, Verona, USA). All study participants provided written informed consent. This study received ethical approval by the Health Research Authority and Health and Care Research Wales (REC reference 21/NW/0023 and UCL-RFH).

### Blood sample collection, L-EV isolation and characterization from plasma

**Fig. 1:**
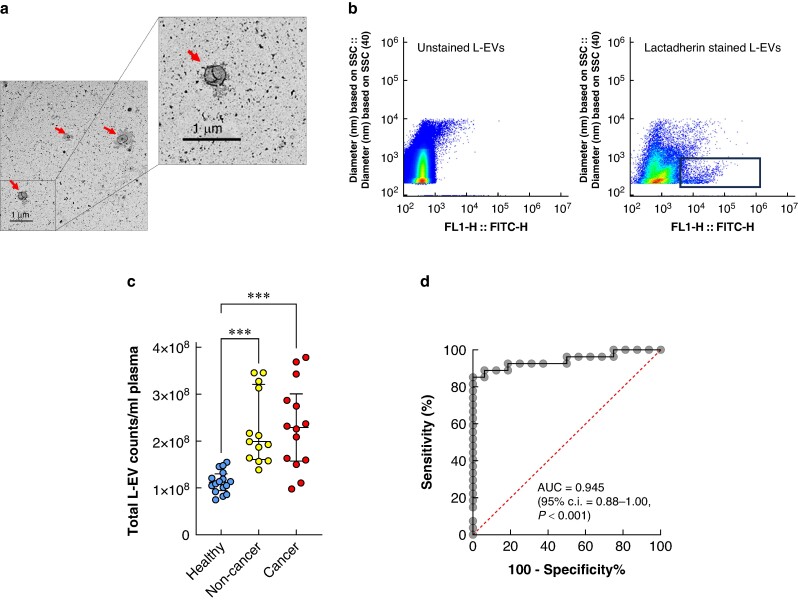
Total L-EV concentrations are elevated in plasma of patients with indeterminate Thy3f thyroid nodules compared with healthy controls **a** Transmission electron microscopy images demonstrating the morphology and size of L-EVs (100–1000 nm). **b** Pseudocolour plot of unstained (left) and lactadherin-FITC stained (right) L-EV samples; calibrated violet side scatter on the *y*-axis (diameter in nanometres) and FITC-H on the *x*-axis (arbitrary flow cytometry units). A size gate ranging from the limit of detection around 210 nm to an upper boundary of 1 µm. **c** The concentration of total plasma L-EV counts stained with lactadherin-FITC. Healthy controls (*n* = 16): blue circles, non-cancer Thy3f nodules: (*n* = 14) yellow circles and cancer Thy3f nodules: (*n* = 14) red circles. **d** A receiver operator curve (ROC) analysis of total lactadherin-FITC staining L-EV concentrations in indeterminate Thy3f thyroid nodules compared with healthy controls. Area under the curve (AUC) of 0.945 (95% c.i. 0.88 to 1.00, *P* = 0.001). Statistical analysis was tested using the Kruskal–Wallis test. Statistical significance was set at *P* ≤ 0.05 (two-sided). Data are presented as median and interquartile ranges. ***P < 0.001. SSC, side scatter; L-EV, large extracellular vesicle; FITC, fluorescein isothiocyanate.

Blood samples were collected in lavender EDTA vacutainer tubes (BD, Oxford, UK) from Thy3f patients after an overnight fast, immediately before undergoing surgery and before the induction of anaesthesia. Blood samples from HCs were similarly collected after an overnight fast. Within 2 h of blood draw, platelet poor plasma (PPP) was obtained by double centrifugation: first at 56,112 r.p.m/2,350 rcf for 10 min to obtain plasma, then 81,966 r.p.m./5,000 rcf for 10 min to deplete platelets (MIBlood EV checklist^16^ with details of blood handling for L-EV isolation are in *Supplementary file S2*). PPP was aliquoted then stored at −80°C until analysis. L-EVs were isolated by differential centrifugation, size exclusion chromatography (SEC) or the Qiagen ExoRNEasy midi kit (Qiagen, Venlo, Netherlands) for analysis by flow cytometry, mass spectrometry and microRNA (miRNA) sequencing respectively. L-EV morphology and size distribution were assessed by transmission electron microscopy (*Fig. 1a*) and nanoparticle tracking analysis (*Supplementary file S1, Fig. S5*) respectively. The potential lipoprotein contamination level was measured by western blotting using anti-apolipoprotein B (*Supplementary file S1, Fig. S7*).

### Measurement of L-EV surface markers by flow cytometry

L-EVs were analysed using a scatter calibrated Cytoflex S flow cytometer (Beckman Coulter, USA) with violet (405 nm laser) side scatter. The size gate was set between approximately 210 (limit of detection) to 1000 nm. L-EVs expressing the anionic phospholipid phosphatidylserine were labelled with the generic EV marker lactadherin conjugated to fluorescein isothiocyanate (FITC) (BLAC-FITC, Prolytix, Vermont, USA)^17^. L-EVs positive for lactadherin and within size range 210–1000 nm were assessed for positivity to different surface protein markers by incubation with appropriate antibody-fluorophore conjugates (*Fig. 1b*). EpCAM-BV421 was studied as an epithelial marker, while surface cancer markers analysed were atypical chemokine receptor type 7 conjugated to phycoerythrin (CXCR7-PE), extracellular matrix metalloproteinase inducer conjugated to brilliant violet 421 (CD147-BV421) and syndecan-4 conjugated to allophycocyanin (SDC4-APC). Antibodies were purchased from BD Biosciences, USA, except for SDC4 which was from R&D (Bio-Techne, Abingdon, UK). Detailed L-EV flow cytometry methods and the MIFlowCyt-EV guidelines^18^ checklist are in *Supplementary file S1, Fig. S2*.

### L-EV miRNA profiling by next generation sequencing

Plasma L-EVs from six non-cancer patients (two hyperplastic nodules, three follicular adenomas, one non-invasive follicular thyroid neoplasm with papillary like nuclear features (NIFTP)), eight cancer patients (five follicular variant of papillary thyroid carcinoma (FV-PTC), one mixed classic and FV-PTC, two follicular thyroid carcinoma (FTC)), and three age, sex and BMI matched HCs (pooled from seven HCs) were sequenced. The integrity and concentration of RNA were assessed using an Agilent 2100 Bioanalyzer (Agilent technologies, USA) (*Fig. S3*). BGI Genomics performed miRNA library preparation using an in-house kit and next generation sequencing (NGS) was performed on the DNBSEQ-G400 platform (BGI, Hong Kong). Reads were aligned to the reference genome: Homo_sapiens_UCSC_hg38 (RefSeq & Gencode gene annotations)^19^.

### L-EV proteome profiling by liquid chromatography/mass spectrometry

After L-EV isolation by a combination of differential centrifugation and SEC, L-EV-rich fractions with minimal plasma protein contamination were identified by flow cytometry and total protein measurements using the BCA Protein Assay Kit (23227, Thermofisher Scientific, USA) (details in *Supplementary file S1, Fig. S4*). Plasma L-EVs from 12 non-cancer Thy3f patients (5 hyperplastic nodules, 4 follicular adenomas, 3 NIFTPs), 12 cancer patients (6 FV-PTC, 3 mixed classic and FV-PTC, 3 FTC), and ten age, sex and BMI matched HCs were digested by trypsin and prepared for label-free quantification liquid chromatography/mass spectrometry (LC/MS). Samples were analysed in duplicate on a Bruker timsTOF Pro mass spectrometer connected to an Evosep One liquid chromatography system (Evosep, Denmark) as detailed in *Supplementary file S1*. Raw data was searched against the *Homo sapiens* subset of the Uniprot Swissprot database. We have submitted all relevant data of our experiments to the EV-TRACK knowledgebase (EV-TRACK ID: EV240166)^20^.

### Statistical analysis

Data were analysed using GraphPad Prism version 10 (GraphPad Software, San Diego, CA, USA). Depending on data distribution, data were expressed as median and interquartile range if not normally distributed or mean and standard deviation if normally distributed. For inference statistics, *t* test, ANOVA or their non-parametric alternatives were used to analyse continuous data as appropriate. The chi-square test was used to analyse categorical data. Receiver operating characteristic (ROC) curve and area under the curve (AUC) analyses were used to establish the diagnostic accuracy of L-EVs. Statistical significance was regarded when *P* value ≤ 0.05 (two-sided). For NGS data, expression of miRNAs was compared between patients with cancer, non-cancer nodules and HCs using DeSeq2 software on the Dr Tom bioinformatics data analysis portal (BGI, Hong Kong). Mass spectrometry data was analysed by GraphPad Prism and Perseus software^21^ and Venn diagrams created in Venny^22^. Significant differential expression of miRNAs and proteins was set at *P* ≤ 0.05 and a log2 fold change threshold −1 > log2FC > 1^23^. An *a priori* power calculation was not possible since this is, to our knowledge, the first study looking at L-EVs as diagnostic biomarkers for Thy3f nodules. Post-hoc power calculation for L-EV counts from flow cytometry were conducted and the statistical power was more than 95% for total L-EV counts performed using G*Power (version 3.1, website: http://www.gpower.hhu.de/).

For dichotomizing Thy3f diagnoses, the low-risk tumour NIFTPs were analysed in the non-cancer arm.

## Results

### Patient characteristics

A total of 42 patients with Thy3f nodules and 16 HCs were included in this study. The final postoperative histopathological diagnoses of the patients were benign in 22: hyperplastic nodules in 10 of 22 (45%), follicular adenoma in 8 of 22 (36%), NIFTP in 4 of 22 (18%); 20 (48%) patients had thyroid cancer. A summary of patient and tumour characteristics is presented in *Table 1* and *Table S1*.

**Table 1:** Demographics of patients with Thy3f nodules and healthy individuals, concentrations of total L-EVs and L-EV subpopulations expressing cancer markers

	Age (years), median (i.q.r.)	Sex (M : F)	BMI (kg/m^2^), median (i.q.r.)	Nodule size (mm), median (i.q.r.)	Total L-EV/ml, median (i.q.r.)	CXCR7 L-EV/ml, median (i.q.r.)	CD147 L-EV/ml, median (i.q.r.)	SDC4 L-EV/ml, median (i.q.r.)	EpCAM L-EV/ml, median (i.q.r.)
Healthy controls (*n* = 16)	48 (25–75)	6:10	24.3 (21.2–33)	–	1.12 × 10^8^ (0.95–1.30 × 10^8^)	1.01 × 10^8^ (0.86–1.16 × 10^8^)	1.51 × 10^8^ (1.27–1.91 × 10^8^)	1.36 × 10^8^ (1.20–1.80 × 10^8^)	3.45 × 10^7^ (2.56–4.24 × 10^7^)
Non-cancer Thy3f thyroid nodules (*n* = 14)	49.5 (24–73)	6:8	24.97 (20.5–38.2)	28 (4–60)	1.99 × 10^8^ (1.61–3.21 × 10^8^)	1.82 × 10^8^ (1.46–2.90 × 10^8^)	2.30 × 10^8^ (1.83–2.76 × 10^8^)	2.16 × 10^8^ (1.74–2.56 × 10^8^)	5.57 × 10^7^ (4.40–6.69 × 10^7^)
Cancer Thy3f thyroid nodules (*n* = 14)	46.3 (28–72)	4:10	25.3 (23.1–28)	19 (2–56)	2.29 × 10^8^ (1.58–3.79 × 10^8^)	2.09 × 10^8^ (1.44–2.74 × 10^8^)	2.28 × 10^8^ (1.51–2.94 × 10^8^)	2.37 × 10^8^ (1.64–2.95 × 10^8^)	5.57 × 10^7^ (4.55–8.90 × 10^7^)
*P* value (ANOVA)	0.256	0.583	0.325	0.389	< 0.001	< 0.001	0.017	0.006	< 0.001

i.q.r., interquartile range; HC, healthy control; L-EV, large extracellular vesicle; cancer markers: epithelial marker: EpCAM, epithelial cell adhesion molecule; cancer markers: CXCR7, atypical chemokine receptor type 7; CD147, extracellular matrix metalloproteinase inducer; SDC4 syndecan-4.

### Total and subpopulations of plasma L-EVs are elevated in patients with indeterminate thyroid nodules *versus* healthy controls

The concentrations of total plasma L-EVs were significantly higher in patients with cancerous and non-cancer Thy3f nodules compared with HCs (*P* < 0.001). Total plasma L-EV concentrations could differentiate patients with Thy3f nodules from HCs with AUC = 0.9444 (95% c.i. 0.8759 to 1.000, *P* < 0.001). Sensitivity and specificity were 92.6% (95% c.i. 76.63 to 98.68) and 81.2% (95% c.i. 56.99 to 93.41) respectively at a cut-off of 1.36 × 10^8^ L-EVs/ml (*Fig. 1c, d*).

**Fig. 2:**
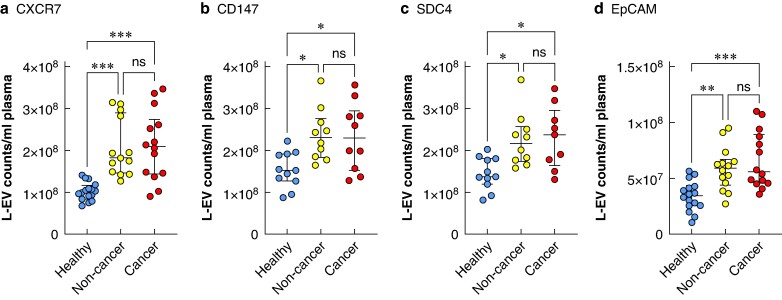
Concentrations of L-EVs expressing CXCR7, CD147, SDC4 and EpCAM are elevated in plasma of patients with indeterminate Thy3f thyroid nodules compared with healthy controls. L-EVs expressing: **a** CXCR7, **b** CD147, **c** SDC4 and **d** EpCAM measured in patients with cancer and non-cancer Thy3f nodules and healthy controls. Statistical analysis was tested using the Kruskal–Wallis test. Statistical significance was set at *P* ≤ 0.05 (two-sided). Data are presented as median and interquartile ranges. ***P < 0.001, **P < 0.01, *P ≤ 0.05, ns *P* > 0.05. L-EV, large extracellular vesicle; CXCR7, atypical chemokine receptor type 7; CD147, extracellular matrix metalloproteinase inducer; SDC4, syndecan-4; EpCAM, epithelial cell adhesion molecule; ns, non significant.

Plasma concentrations of L-EVs expressing surface markers CXCR7 (*P* < 0.001), CD147 (*P* = 0.001), SDC4 (*P* = 0.03) and EpCAM (*P* < 0.001) were higher in patients suffering from both benign and malignant Thy3f nodules compared with HCs (*Fig. 2a–d*). Total and subpopulation concentrations of plasma L-EVs did not discriminate between non-cancer and cancer Thy3f nodules.

### Differentially expressed circulating L-EV miRNAs in patients with cancerous compared with non-cancerous Thy3f nodules

**Fig. 3:**
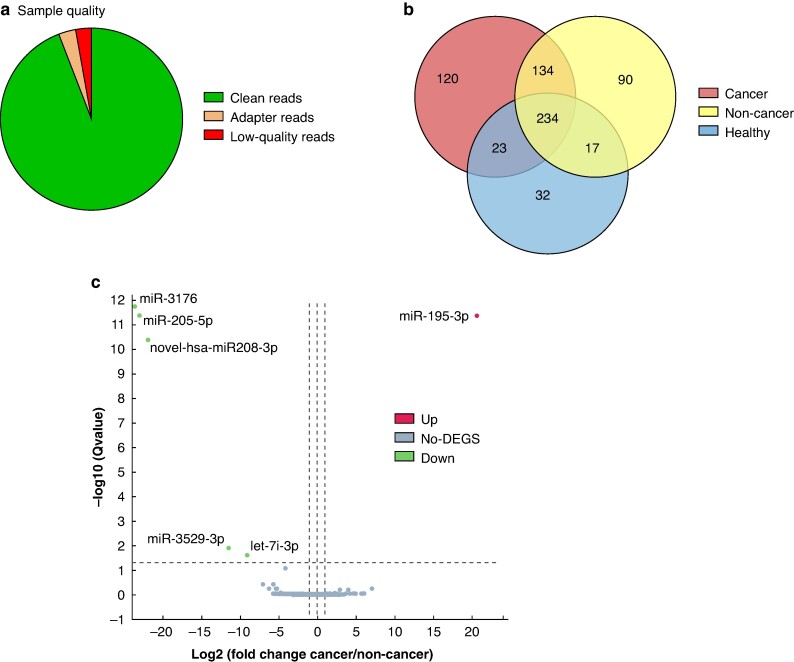
L-EV miRNA sequencing **a** Quality of plasma L-EV miRNA next generation sequencing showing ∼95% clean reads from total sequenced reads. **b** Intersections between miRNAs identified in healthy controls (blue), patients with non-cancer (yellow) and cancer (red) Thy3f nodules are represented in the Venn diagram; the numbers represent the miRNAs identified in the respective group. **c** Volcano plot of differentially expressed miRNAs in patients with cancer *versus* non-cancer Thy3f nodules. The criteria for significant differential expression in DESeq2 was set at false discovery rates <0.05 and a fold change threshold, −1 > log2FC > 1. All miRNA sequencing data was analysed on the Dr Tom data analysis portal (BGI, Hong Kong). DEGS, differentially expressed genes/micro-RNAs; L-EV, large extracellular vesicle; miRNA, microRNA.

NGS of EV miRNAs yielded an average 24.12 million reads per sample. The average alignment ratio to the reference human genome was 60.99%. Percentage of clean tag reads ranged from 74.96 to 93.54%. NGS detected 650 miRNAs across all 20 samples; 120 miRNAs were exclusive to Thy3f cancer patients, 90 were exclusive to Thy3f non-cancer patients and 32 exclusive to HCs. A total of 234 miRNAs were common to all groups (*Fig. 3a, b*).

Comparing cancer *versus* non-cancer Thy3f nodule patients, six miRNAs were significantly differentially expressed. One was upregulated, mir-195-3p (log2FC = 20.7, *P* < 0.001), and five were downregulated: mir-3176 (log2FC = −23.6, *P* < 0.001), mir-205-5p (log2FC = −23.0, *P* < 0.001), novel-hsa-mir-208-3p (log2FC = −21.9, *P* < 0.001), mir-3529-3p (log2FC = −11.5, *P* = 0.01) and let-7i-3p (log2FC = −9.1, *P* = 0.02) (*Fig. 3c*). Principal component analysis of miRNA sequencing data is shown in *Fig. S1*. Comparisons between Thy3f groups and HCs are in *Fig. S6* and *Table S2*, and mRNA targets for differentially expressed miRNAs between patients with cancer/non-cancer Thy3f nodules in *Figs S8*–*12*.

### Differentially expressed circulating L-EV proteins in patients with cancerous compared with non-cancerous Thy3f nodules

**Fig. 4:**
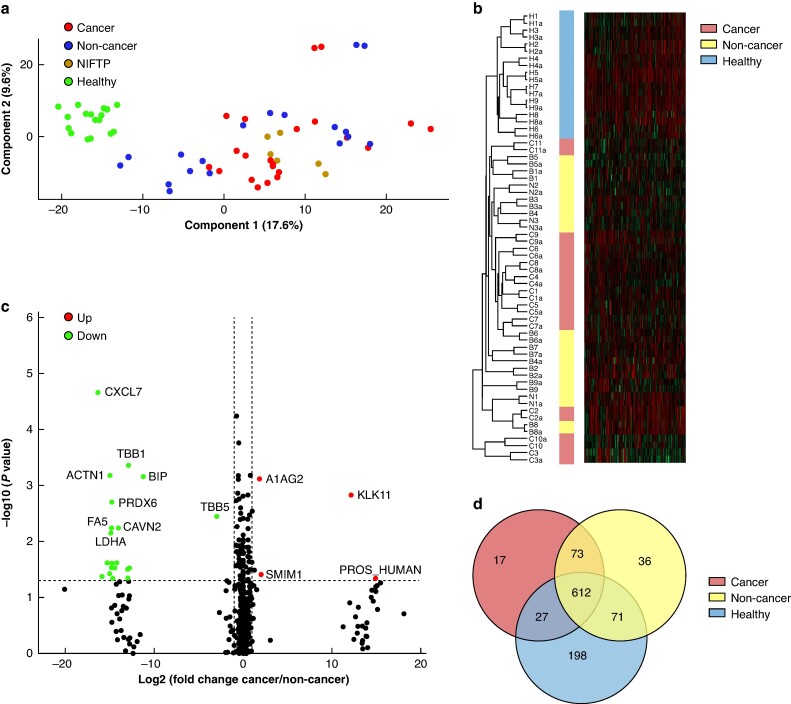
L-EV proteomics **a** Principal component analysis clustering samples based on their protein expression profiles. **b** Heatmap generated by unsupervised hierarchical clustering depicting a colour scale reflecting protein abundance. Horizontal rows indicate 34 independent L-EV samples (12 cancer and 12 non-cancer Thy3f samples, and 10 HCs). Vertical columns indicate the proteins identified by mass spectrometry (*n* = 1034 proteins). **c** Volcano plots of the differentially expressed proteins in L-EVs of patients with cancer compared with non-cancer Thy3f nodules. Statistical significance was tested using the Kruskal–Wallis test *P* < 0.05 and a fold change threshold, −1>|log2FC|> 1. **d** Venn diagram demonstrating intersections between proteins identified in healthy controls (blue), non-cancer (yellow) and cancer (red) Thy3f nodules, representing the number of proteins identified within the respective groups. PCA and heatmap were generated using Perseus software^21^, and Venn diagrams were created in Venny (https://bioinfogp.cnb.csic.es/tools/venny/). NIFTP, non-invasive follicular thyroid neoplasm with papillary like nuclear features; HC, healthy control; L-EV, large extracellular vesicle; PCA, principal component analysis.

A total of 1034 proteins were identified across all 32 samples. From the top 20 EV proteins reported in Vesiclepedia, an online EV molecular data repository^24^, 15 were identified in our samples. The top five are: β-actin, flotillin 1, annexin A2, tetraspanin CD9 (CD9), and tetraspanin CD81 (CD81). Only β-actin was expressed across all 32 samples, while CD81 was expressed in the least number of samples (24 of 32). Principal component analysis (*Fig. 4a*) and unsupervised hierarchical clustering (*Fig. 4b*) showed HCs tightly clustered, while cancerous and non-cancer Thy3f nodules are interspersed. The Venn diagram (*Fig. 4d*) demonstrates 17 proteins expressed only in Thy3f cancer patients, 36 in Thy3f non-cancer patients, while 198 were exclusive to HCs; 612 proteins were common to all groups. Differential expression analysis showed three proteins significantly overexpressed in cancerous Thy3f nodules compared with non-cancer: kallikrein-related peptidase 11 (KLK11) (log2FC = 12; *P* = 0.001), α-1-acid glycoprotein 2 (A1AG2) (log2FC = 2; *P* = 0.007) and small integral membrane protein 1 (SMIM1) (log2FC = 2, *P* = 0.04). Significantly underexpressed were 20 proteins. The most underexpressed were chemokine (C-X-C motif) ligand 7 (CXCL7), tubulin β-1 chain (TBB1), actinin α-1 (ACTN1) and binding immunoglobulin protein (BIP) (*Fig. 4c*).

## Discussion

This study explored the potential of circulating L-EVs as surrogate diagnostic biomarkers for indeterminate Thy3f thyroid nodules. Two novel findings are, first, a multi-platform omics approach identified several candidate L-EV miRNA and protein biomarkers that could potentially distinguish between patients with non-cancer and cancer Thy3f nodules. With respect to the cancer group, the upregulation of mir-195-3p, KLK11, A1AG2, SMIM1, and the downregulation of mir-3176, mir-205-5p, novel-hsa-mir-208-3p, mir-3529-3p and let-7i-3p, CXCL7, TBB1, ACTN1 and BIP were identified compared with the non-cancer group. Second, the concentrations of total and subpopulations of tumour-related plasma L-EVs were significantly higher in all patients with Thy3f nodules compared with HCs, with a high AUC. Similar findings have been shown in our laboratory where total L-EV concentrations were higher in benign and malignant colorectal polyps compared with HCs and are potential biomarkers for colorectal cancer screening and diagnosis^25^.

To our knowledge, this is the first study investigating circulating EVs as diagnostic biomarkers of Thy3f nodules. Previous literature mainly focused on circulating small extracellular vesicles/exosomes (sEVs) as diagnostic or prognostic biomarkers for PTC^26-32^. Unlike Thy3f nodules, PTCs can be confidently diagnosed on cytopathology in most patients based on their nuclear features^1^. Moving forward, the identified biomarkers from this study will serve as candidates for validation on a larger patient cohort using clinically established techniques such as polymerase chain reaction (PCR) and enzyme-linked immunosorbent assay (ELISA) for miRNA and proteins respectively. These techniques are in routine use in clinical laboratories, thus not needing specialized or centralized facilities. Furthermore, sEVs rather than L-EVs were the focus of previous research in the context of thyroid cancer biomarkers^26–32^. L-EV isolation is easier than sEVs, which require ultracentrifugation at very high speeds, whereas L-EVs can be isolated by benchtop centrifuges at vastly lower speeds. In addition, L-EVs potentially carry a broader range of cargo molecules, owing to their larger size, as well as representative membrane markers, owing to their cell membrane origin^33^.

Previous studies of sEVs showed that a panel of five miRNAs could discriminate follicular adenoma (FA) from FTC with an AUC of 0.924^34^, while another study^35^ identified two miRNAs of the Let-7 family (Let-7f and Let-7d) to be overexpressed in a subgroup of plasma sEVs expressing thyroid peroxidase (TPO) that potentially can discriminate FA from FTC^35,36^. Despite this, these studies only compared FA and FTC, thus these findings may not translate to clinical practice as this dichotomy does not represent the differentials of Thy3f nodules’ final histopathology, which also include hyperplastic nodules, FV-PTCs (the most common benign and malignant diagnoses of Thy3f nodules) and NIFTPs^37^.

In this work, hsa-mir-195-3p was significantly upregulated in plasma L-EVs of Thy3f cancer patients, similar to previous work showing its upregulation in serum of patients with well differentiated thyroid cancer (WDTC) compared with FA^38^. Paradoxically, expression levels of hsa-mir-195-3p were downregulated in WDTC tissue compared with FA^38^. Together with our findings, this may indicate that hsa-mir-195-3p is selectively packaged and secreted from thyroid cancer cells into the bloodstream enclosed in EVs. Given its demonstrated pro-tumourigenic roles in cancer signalling pathways, including retinoblastoma-early region 2 binding factor and phosphatidylinositol 3-kinase pathways^39^, hsa-mir-195-3p has strong potential as a relevant diagnostic biomarker for cancer in patients with Thy3f nodules.

KLK11 was significantly overexpressed in circulating L-EVs of Thy3f cancer compared with non-cancer patients. KLK11 has been implicated in carcinogenesis and is upregulated in ovarian, prostate, lung and rectal cancers^40^. In a recent study, KLK11 mRNA levels were reported to be overexpressed in thyroid cancer compared with adjacent normal thyroid tissues, while its silencing in PTC cell lines inhibited proliferation, migration and invasiveness^41^.

Circulating L-EVs from Thy3f cancer patients had higher protein expression of A1AG2. Like the related A1AG1, it is an acute-phase plasma protein predominantly expressed in hepatocytes and upregulated in cancers including thyroid, hepatocellular and colorectal^42–44^. Recently, A1AG2 was identified by mass spectrometry as one of the top three upregulated proteins in PTC compared with normal thyroid tissues^42^. This protein has also been previously identified in plasma EVs^45^. The related A1AG1 is listed as a plasma and urinary EV protein in ExoCarta, an online EV database^46^. Another upregulated L-EV protein in patients with cancer over non-cancer Thy3f nodules is SMIM1, a conserved small protein with a role in red blood cell development^47^. A study characterizing EVs from 60 cell lines^48^ detected SMIM1 in EVs isolated from eight cancer cell lines, as well as in EVs isolated from human plasma^49^, breast milk and seminal fluid^24^. SMIM1 is overexpressed in many cancers, with thyroid cancer showing the highest expression by immunohistochemistry^50^. Interestingly, in the present study, β-actin was the only protein expressed in all L-EV samples as identified by proteomics and thus may serve as a generic marker for L-EVs.

This study exhibits many strengths that underscore its significance. Noteworthy attributes include its focus on a critical clinical matter, the use of clinical patient samples, a well matched HC group, well established methods of isolating and analysing L-EVs, and the identification of promising candidate L-EV biomarkers with the potential to distinguish between cancer and non-cancer Thy3f nodules. A liquid biopsy that could identify cancerous nodules without the need for diagnostic surgery would be of significant clinical relevance^1^. This would help reduce exposure to undue risks and potential complications of diagnostic surgeries. L-EVs are a promising diagnostic modality that can be translated to clinical use owing to their stability and relative ease of isolation^25^.

Despite its strengths, it is important to acknowledge that this study is in its initial discovery phase and the sample size remains relatively small. This is not unprecedented in omics feasibility studies that are usually tailored to identify potential biomarkers for further validation^31,32,51^. The variety of histological subtypes of nodules included might introduce heterogeneity to this study, which may limit the power of conclusions drawn. However, excluding some Thy3f nodules’ differential diagnoses may introduce a selection bias not reflective of the range of diagnoses. Further work will require larger cohorts accounting for the breadth of Thy3f histological outcomes. Additionally, L-EV biomarkers were measured in total circulating L-EVs which are indeed a pool shed from all body organs^52^, thus some L-EVs may not be thyroid tumour derived. As such, further work is required to investigate the underlying biology of the identified markers. However, biomarkers may originate from cancer cells, cells in the tumour microenvironment (such as stromal cells, blood vessels, immune cells) or as a result of the host’s immune response to cancer or other systemic processes triggered by the presence of the cancer^52–54^. Hence specific L-EV biomarkers that are consistently differentially expressed in Thy3f cancer patients could still be used as a tumour fingerprint^53^ and remain valuable even if not directly derived from cancer cells^31^. This is reinforced by the fact that the only EV liquid biopsy-based cancer diagnostic tests that have reached clinical application use total EVs including ExoDx™ Lung (ALK) and ExoDx Prostate IntelliScore (EPI), which analyse mRNAs extracted from total EVs as biomarkers for non-small cell lung cancer and prostate cancer respectively, with high sensitivity and specificity^55,56^. In terms of costs, the miRNA and protein profiling of plasma L-EVs currently stand at a fraction of the cost of the commercially available molecular tests of fine needle aspiration material^57^. As the test moves into the validation phase with clinically established techniques, a much greater cost reduction is anticipated.

## Supplementary Material

zrae139_Supplementary_Data

## Data Availability

The data that support the findings of this study are available on request from the corresponding author.
