# Supplementary material for: Circulating large extracellular vesicles as diagnostic biomarkers of indeterminate thyroid nodules: multi-platform omics analysis
Source: BJS Open. 2024 Dec 30;9(1):zrae139. doi: 10.1093/bjsopen/zrae139 (PMC11683363; doi:10.1093/bjsopen/zrae139)
Supplement: zrae139_Supplementary_Data [file zrae139_supplementary_data.zip › BJS5-2024-07-0279.R1 CLEAN BJS-Open-supplementary-material-template-SS-CS00039106-2.docx]

**Circulating Large Extracellular Vesicles as Diagnostic Biomarkers of Indeterminate Thyroid Nodules: Multi-Platform Omics Analysis**

Authors: Nada M Ahmed^1,2^, Mohammad MR Eddama^3^ MD PhD FRCS, Kevin Beatson^3^ MBChB MRCS, Rijan Gurung^3^ PhD, Jigisha Patel^3^ PhD, George Iskandar^5^ MD FRCA, Alaa Abdel-Salam^4^ FRCS, Abdullah Al-Omar^4^ FRCS, Richard Cohen^3^ MD FRCS, Tarek Abdel-Aziz^4, ±^ MD FRCS, Lucie Clapp^1, ±^ PhD.

^1^ Institute of Cardiovascular Sciences, University College London; London, UK.

^2^ Pathology Department, Alexandria University, Alexandria, Egypt

^3^ Research Department of Surgical Biotechnology, Division of Surgery and Interventional Science, University College London; London, UK.

^4^ Endocrine Surgery Unit, University College London Hospitals, London, UK

^5^ Department of Anaesthesia and Perioperative Medicine, University College London Hospitals, London, UK

^±^ Shared senior authorship.

**Corresponding author**

Mohammad M.R. Eddama MD PhD MSc FRCS FHEA

Principal Research Fellow, Research Department of Surgical Biotechnology, University College London, UK

GI Services, Ground Floor, 250 Euston Road, London NW1 2PG

Tel: +44 (0)7747 061071

Email: [m.eddama.12@ucl.ac.uk](mailto:m.eddama.12@ucl.ac.uk)

**Supplementary Materials - Index**

| **Supplementary Methods** |  |
| --- | --- |
| L-EV isolation methods | *pag. 3* |
| Transmission electron microscopy imaging of L-EVs | *pag. 4* |
| Nanoparticle tracking analysis (NTA) for characterization of size distribution of L-EVs | *pag. 5* |
| Flow cytometry methods-MIFlowCyt-EV framework checklist | *pag. 5* |
| Mass spectrometry methods | *pag. 11* |
| **Supplementary Figures and Tables** |  |
| Figure S1: Principal component analysis plot of the miRNA sequencing data. | *pag. 12* |
| Figure S2: Pseudocolour plot showing PE-fluorescent 3 µm counting beads (MP count beads, Biocytex, Marseille, France | *pag. 13* |
| Figure S3: Quality control of large extracellular vesicles miRNA samples for next generation sequencing. | *pag. 14* |
| Figure S4: Size exclusion chromatography fractions characterisation | *pag.14* |
| Figure S5: NTA analysis of L-EV diameter distribution for a representative pooled and concnetrated SEC L-EV rich fractions 1–4. The median L-EV size was 128nm, mode of 110nm. These measuremnts were made using ZetaView PMX-420 Quatt (Particle Metrix, Germany) equiped with a 488nm laser | *pag.15* |
| Figure S6: Venn diagram depicting the intersections between miRNAs identified in healthy controls (green), non-cancer (orange, left Venn diagram), and cancer (orange, right Venn diagram) Thy3f nodules, representing the number of miRNAs identified within the respective groups | *pag.15* |
| Figure S7: Representative western blot image | *pag.16* |
| Figure S8: KEGG pathway analysis of the mRNA targets for hsa-miR-195-3p | *pag.17* |
| Figure S9: KEGG pathway analysis of the mRNA targets for hsa-mir-3176 | *pag.18* |
| Figure S10: KEGG pathway analysis of the mRNA targets for hsa- mir-205-5p | *pag.19* |
| Figure S11: KEGG pathway analysis of the mRNA targets for novel-hsa-mir-208-3p | *pag.20* |
| Figure S12: KEGG pathway analysis of the mRNA targets for hsa- mir-3529-3p | *pag.21* |
| Figure S13: KEGG pathway analysis of the mRNA targets for hsa- let-7i-3p | *pag.22* |
| **Table S1** | *pag.23* |
| **Table S2** | *pag.28* |
| **References** | *pag.33* |

**Supplementary Methods**

# L-EV isolation methods

**For flow cytometry:** Frozen platelet poor plasma (PPP) (100µl aliquot from each sample) were thawed in a 37°C water bath for 1 minute. L-EVs were isolated from PPP by centrifugation at 16,000g/rcf for 1 hour at 4^o^C to pellet L-EVs. The supernatant was decanted, and the pellet was left to air dry by inverting the tubes on a chromatography paper. Then the L-EVs were resuspended in 175 µls of 0.1 µm filtered PBS (filtered by sterile Millex-VV Syringe Filter Unit with a pore size 0.1 μm (SLVVR33RS, Merck Millipore, USA)). The pellet was thoroughly resuspended by mixing and pulse vortexing, avoiding bubble formation. Then the resuspended pellet was divided into 35μl aliquots and plated into a 96 well U-bottomed polypropylene plate (Greiner, Sigma Aldrich, Gillingham, UK).

**For L-EV miRNA next generation sequencing:** EVs were isolated, and RNA extracted from 1 ml of PPP using ExoRNeasy midi kit (Qiagen, Hilden, Germany, 77144) following the manufacturer’s instructions. Briefly, L-EVs from 1ml of PPP were captured on membrane affinity spin columns supplied in the kit, which capture L-EVs on the membranes by their phospholipid bilayer membrane. This is to exclude non-vesicular miRNAs circulating in the plasma (e.g. carried on plasma proteins) which will pass through the membrane and are eluted, leaving only L-EVs attached to the columns’ membranes. Then total RNA is extracted by Qiazol which is provided with the kit. A modification was made to the manufacturer’s protocol to maximise yield and purity of the RNA extracted. Qiagen MaXtract High Density 2mL phasemaker tubes (Qiagen, Hilden, Germany, 129056) were used to allow precise separation of the upper aqueous phase containing the RNA from the rest of the phases during RNA extraction.

**For L-EV proteomics:** L-EVs were isolated using a combination of centrifugation and size exclusion chromatography (SEC). The reason for combining 2 isolation methods is to obtain a purer L-EVs preparation compared to either method spearately. This is crucial to minimise the contamination of the L-EV proteins with the much more abundant plasma proteins which are known to overshadow and shift the dynamic range of mass spectrometry machines, limiting the detection of the less abundant but relevant L-EVproteins of interest (1). First, 1ml of PPP was centrifuged at 17,000 g/rcf for 1 hour at 4^o^C, the supernatant was decanted, and the pellet was left to air dry by inverting the tubes on a chromatography paper. Then the L-EV pellets were thoroughly resuspended by mixing and vortexing in 160µl of 0.1µm filtered PBS. This was followed by SEC using qEVsingle Gen2 70nm pore size Columns (Izon, New Zealand, ICS-70). These columns allow the separation of L-EVs which are 70-1000nm in size from other smaller particles (contaminating plasma proteins will have a smaller size and will be separated from the L-EV fractions). A new column was used for each sample to avoid any carry over or cross-contamination between samples. L-EV isolation by SEC was done by adding 150 microliters of the resuspended L-EVs onto the SEC columns and SEC was performed according to manufacturer’s instructions (https://4136435.fs1.hubspotusercontent na1.net/hubfs/4136435/Manuals%20Technical%20Notes%20and%20Customer%20Support/qEV%20columns/qEVsingle-gen-2-user-manual-ICS-DQ-001.pdf, accessed on 12/03/2024). Briefly, Initially SEC columns were washed by adding 6 mLs of 0.1µm filtered PBS onto the column following manufacturer’s instructions. This was followed by adding 150 µls of the resuspended L-EVs (in 0.1µm filtered PBS) onto the SEC columns. after adding the sample, 170 µls of PBS were added to bring the sample down through the column. Then SEC fractions were collected by sequentially adding 170 µls aliquots of PBS 13 times to the column to collect 13 fractions (160 µls each) per sample. All the 13 fractions were then tested for their L-EV concentrations and total protein content by flow cytometry and Bicinchoninic Acid (BCA) protien assay respectively. Fractions 1-4 were identified to be L-EV rich fractions as they had: 1- the highest counts of total phosphatidylserine expressing L-EVs as measured by flow cytometry and, 2- free of measurable plasma protein contamination as measured by BCA Protein Assay Kit (23227, Thermofisher Scientific, USA). Because SEC yields diluted EVs (2) there was a need to concentrate the purified L-EVs for mass spectrometry analysis. L-EV concentration was performed by adding the L-EV rich fractions (680µl in total of pooled fractions 1-4, 170µl per fractions) from each sample onto a fresh Amicon Ultra 2ml centrifugal filter with a 10 kilo Dalton molecular weight cutoff membrane (UFC201024, Merck Millipore, USA) and concentration was performed according to the manufacturer's instructions(<https://www.sigmaaldrich.com/deepweb/assets/sigmaaldrich/product/documents/406/231/pr05484-rev0219.pdf>, accessed on 12/03/2024). These yield L-EVs concentrated in around 45 µls of PBS.

The similarities and differences between size exclusion chromatography (SEC) and Qiagen ExoRNEasy midi kit (Qiagen, Venlo, Netherlands) which were used for mass spectrometry and miRNA sequencing respectively is as follows:

1- **Similarities**: both L-EV isolation techniques apply further purification of the plasma L-EVs to get rid of contaminating plasma proteins and lipoproteins yielding a purer L-EV preparation compared to differential centrifugation on its own.

2- **Differences**: SEC, as can be appreciated from the name, separates L-EVs from other contaminants based on their size. Excluding the much smaller plasma proteins and lipoproteins yielding a purer L-EV sample that is required for mass spectrometry analysis. Similarly, the exoRNEasy midi kit (Qiagen, Venlo, Netherlands), which was used for miRNA sequencing, also yields a pure L-EV preparation. This method easily isolates L-EVs and separates them from contaminants by capturing the L-EVs by their plasma membranes (i.e. membrane affinity) on a column. Subsequently RNA is extracted from L-EVs by applying a lysis buffer (Qiazol, Qiagen, Venlo, Netherlands) directly to the L-EVs attached to the column membrane. Therefore, the added advantage of this kit for RNA extraction is that it provides a well-established and validated protocol in a combined workflow of L-EV isolation folowed by L-EV RNA extraction.

# Transmission electron microscopy (TEM) imaging of L-EVs

L-EVs isolated by size exclusion chromatography and concentrated using Amicon Ultra centrifugal filters 10kDa (UFC201024, Merck Millipore, USA) (as described above for L-EV isolation methods for L-EV proteomics) were used for TEM imaging. Two µl of L-EVs (suspended in 0.1µm filtered PBS) were placed on a formvar carbon-coated copper grid (TEM-FCF150CU, Merck Sigma-Aldrich, USA) and left for 10 minutes to adsorb. L-EVs were visualised by negative staining using 0.5% uranyl acetate for 2 seconds, followed by washing the grid by placing onto 3 water droplets for 2 minutes each. L-EVs were imaged by Jeol 1400 electron microscope at 120kV (Jeol, Japan).

# Nanoparticle tracking analysis (NTA) for characterization of size distribution of L-EVs

NTA analysis was performed using ZetaView PMX-420 Quatt (Particle Metrix, Germany) equiped with a 488nm laser. L-EVs isolated by SEC were thawed immediately prior to NTA analysis. L-EVs in PBS were further diluted in 0.1µm filtered PBS to the optimal acquisition range of the instrument (100-150 particles) and measured label-free in light scatter mode at a temperature of 22°C using the following settings: sensitivity = 80, shutter = 100, frame rate = 30, trace length = 15, bin size = 5 nm, positions per single reading = 11. Each sample measurement was performed in replicates of 5.

# Flow cytometry methods- MIFlowCyt-EV framework checklist

## Preanalytical variables conforming to MISEV guidelines*

Peripheral venous blood samples were obtained from fasting volunteers (an overnight fast of 7-9 hours). Blood was drawn directly into lavender EDTA 6 mL vacutainer tubes (BD, Oxford, United Kingdom). The blood tubes were always kept upright and transported with minimal agitation in an upright position at room temperature. Within maximum 2 hours, platelet poor plasma (PPP) was prepared from whole blood by a differential (double) centrifugation method. Whole blood in the vacutainer tubes were initially centrifuged at 2350g/rcf for 10 minutes at 20 °C (ALC PK 131 R centrifuge, swing-out rotor, ALC International, Italyname), no brake applied, to separate the plasma. The supernatant (plasma) was collected 5 mm above the buffy coat to avoid platelet contamination. Then the plasma was spun again 5,000 g/rcf for 10 minutes at 20°C with no brake applied (Eppendorf 5415-R centrifuge, fixed angle Rotor 450, Eppendorf). Supernatant (now PPP) was collected 5 mm above the platelet pellet. PPP was pooled and mixed by pipetting up and down gently first to homogenize and avoid inter-aliquot variability, then transferred into 100 µl aliquots in 1.5 mL sterile microcentrifuge tubes, and immediately stored in −80 °C freezer until analysis. For all patients and controls; age, gender, fasting, and smoking status were recorded.

## Experimental design according to MIFlowCyt guidelines*

Aim: To compare the concentration of EVs expressing phosphatidylserine, Epithelial cell adhesion molecule (EpCAM) was studied as an epithelial marker, a panel of surface cancer markers: Atypical chemokine receptor type 7 (CXCR7-PE), Extracellular matrix metalloproteinase inducer (CD147-BV421), and Syndecan-4 (SDC4-APC) in patietns with cancer and non-cancer Thy3f thyroid nodules and healthy controls.

## Sample staining details*

The 100 µl aliquots of PPP were thawed rapidly for 1 min in a 37°C water bath and the aliquots were diluted by equal volume (100 µl) of PBS. This dilution helps to decrease the viscosity of PPP hence increasing the efficiency of L-L-EV sedimentation or pelleting. In addition, this dilution can also increases L-EVs purity by decreasing co-isolated contaminants, such as protein aggregates. Afterwards the PPP was centrifuged at 16,000g for 60 minutes in a refrigerated centrifuge at 4°C (Eppendorf 5415-R centrifuge, fixed angle Rotor Eppendorf AG, Hamburg, Germany), then the supernatant was decanted by inverting the tubes on top of blotting paper and then the pellet left to dry out. The L-EV pellet was then resuspended in 175 µl of 0.1µm filtered PBS, mixed up and down thoroughly and then vortexed. Thirty-five microliters of resuspended L-EVpellet Lactadherin conjugated to fluorescein isothiocyanate (FITC) was diluted in 0.1µm filtered PBS at a 1:10 dilution (final concentration of 8.3μg/mL), and 5μl of that was added to appropriate wells of a 96-well U-bottom multiplate to assess total MV counts. The mixture was incubated for 20 minutes in the dark (by covering with aluminium foil) on a gentle shaker. Each diluted antibody was centrifuged at maximum speed 16,000g for 4 minutes at 4°C to pellet any antibody/florochrome aggregates which can interfere with L-EV analysis.


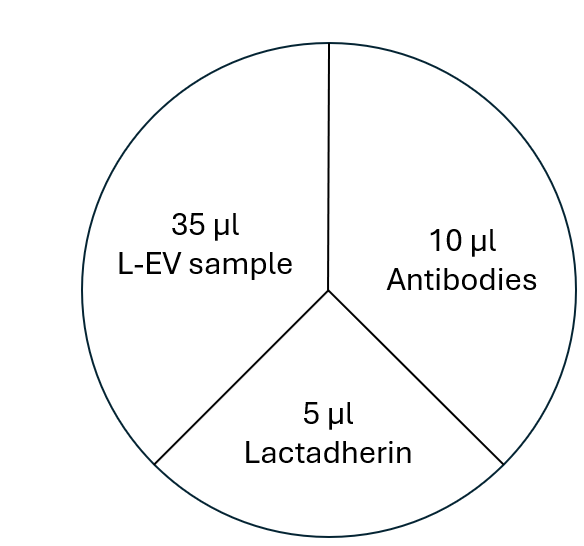


**The constitution of each well of the stained sample.** 5μl

Lactadherin-FITC (1:10 dilution) and 10μl of antibody cocktails were added to 35μl of the sample.

## Sample washing details*

No sample washing step for any excess unbound antibody was performed in the present study, but 180 μl of 0.1 µm filtered PBS was added to each well after the 20 minutes staining to dilute the unbound antibodies and terminate staining.

## Sample dilution details*

All antibodies were titrated before staining L-EVs. This is to get the best possible separation of positive versus negative L-EV populations and avoid too much background. The recommended manufacturers concentration was the starting point that was serially diluted by half onwards to at least 6 points. An unstained sample was always included as a control in all antibody titration experiments to help gate true positive from negative events. Then all the samples were run for the same time and same volume on the flow cytometer with no restriction on event counts as a stopping criterion. Then all the titrations were analysed on Flowjo. The median fluorescence intensity of the positive as well as the negative L-EV populations were plotted against antibody concentrations in µg /ml. Second the stain and separation indices were plotted against antibody concentrations in µg /ml. Finally, event counts of the positive L-EV population were plotted against antibody concentrations in µg /ml. The optimum antibody concentration was chosen as the one showing the highest stain and separation indices, while maintaining acceptably high event count in the positive L-EV population gate. Positive L-EV event counts were always kept at no less than 10,000 events.

## Buffer-only controls*

The flow cytometer was cleaned before every run to render the system as clean as possible and minimise the background noise as much as possible. Buffer only controls were used in every run. 0.1 µm filtered PBS was used in all the experiments and was recorded at the same acquisition settings on the flow cytometer as all L-EV samples. Buffer only controls were used to assess background noise, and set the Violet-SSC threshold and gain. Those were adjusted so that the buffer only control events/second did not exceed 1000 events/second, with an abort rate of less than 1% at the “high event rate” acquisition setting.

## Buffer with reagent controls*

Buffer with reagent controls were included in all the runs and recorded at the same cytometer acquisition settings. Lactadherin and each antibody used in the run was added to PBS in the same concentration used to stain the L-EV samples. This was used to assess whether unbound antibodies can be contributing to the signal. When events were seen in the positive L-EV gate from the buffer and reagent only controls, those were gated out and subtracted from the positive L-EV counts in the actual L-EV samples.

## Unstained controls*

Unstained, pooled L-EV sample control was included in every run to help assess the background autofluorescence of the samples and aid gating of positive versus negative L-EV populations. Also, unstained L-EV samples were used to set the Violet-SSC threshold and gain by adjusting them to allow a maximum of 5000 events/second. Flow cytometer acquisition settings were maintained for L-EV samples and controls.

## Isotype controls**

Isotype controls were not used, instead fluorescent minus one (FMO) controls were used in each run for each fluorochrome used and were used alongside the unstained controls to gate positive L-EV populations.

## Single-stained controls*

Single stained L-EV samples were included in every run. A single stained control for each antibody-fluorochrome was included to assess steric hinderance or competitive inhibition of one antibody with the others and to adjust compensation.

## Procedural controls**

not applicable

## Serial dilutions*

To find a safe sample dilution range where swarming or coincidence events is best avoided, and hence accurate L-EV concentrations can be obtained (3). Ten samples (5 patients, 5 controls) were thawed, vortexed and pooled. 100µl of the pooled samples was then placed in a new sterile 1.5 ml microcentrifuge tube, spun for an hour and then supernatant decanted. The L-EV pellet was then resuspended in 175 µL PBS. Afterwards, the sample was serially diluted 12 times, with 150 µL of sample added to 150 µL of PBS giving a dilution range from 3.16 x which was then serially diluted up to 1000,000 x. The L-EV samples were stained with 5 µL of 1:10 diluted Lactadherin-FITC (previously titrated) measured using a 96-well polypropylene plate (Corning). The 4th, 5th and 6th dilutions showed a linear decrease of measured total event counts across dilutions while maintaining a stable median fluorescence intensities (MFI). The median fluorescence and scatter intensities of all events for dilutions 1 till 3rd were maintained at 500 ± 18 FITC arbitrary units, and 3000 ± 31 side scatter arbitrary units, respectively. After dilution 5 the event counts dropped and their fluorescence and scatter intensity were not maintained. The first serial dilution (1-fold dilution) in the series was therefore used to measure L-EV concentration and report immunostaining data.


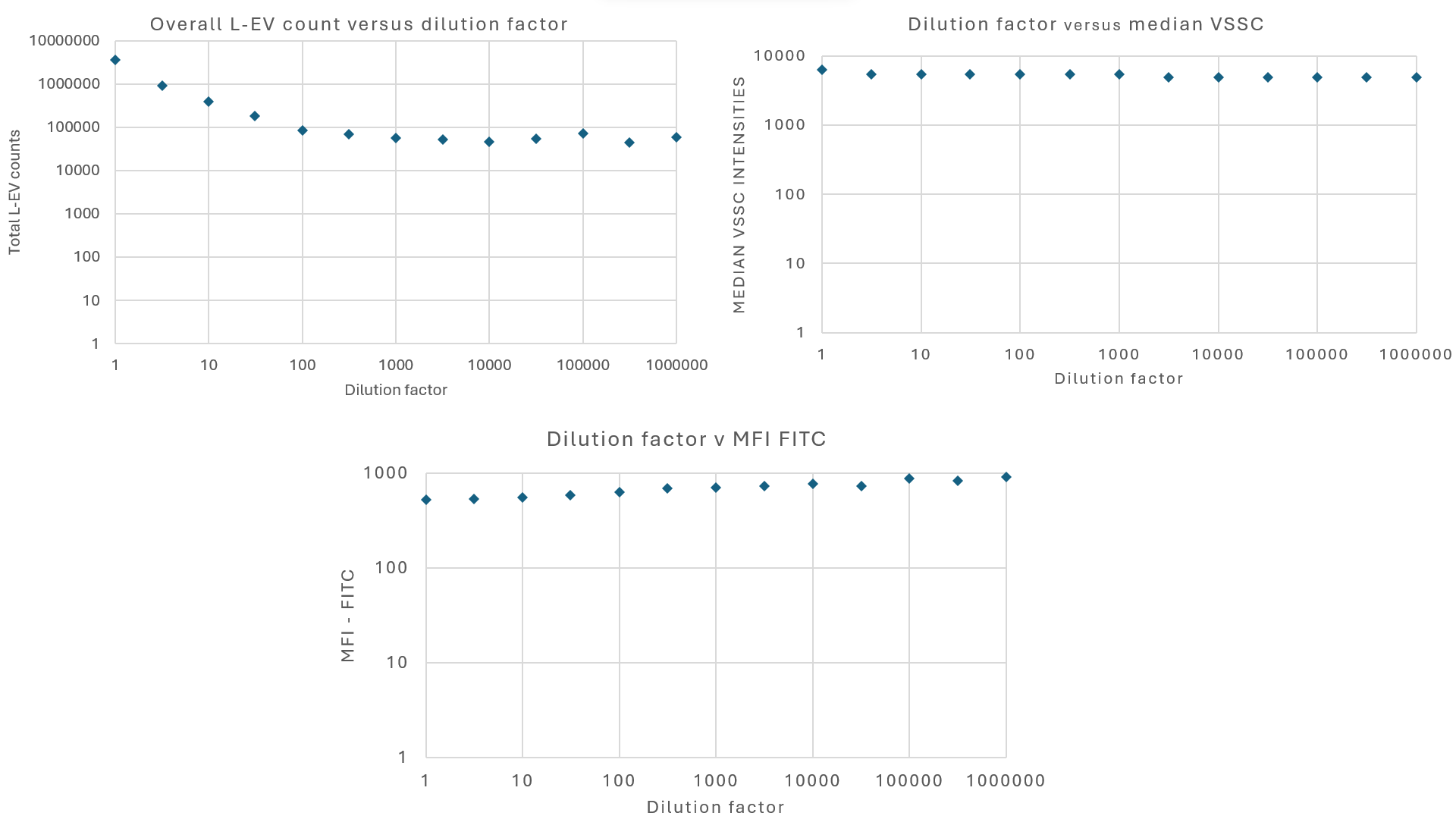


**Serial sample dilution:** samples were diluted over a range from 1x- 1000,000x. **Top left:** total L-EV counts where plotted on the Y axis against the dilution factor on the X axis. There was a gradual decline in the L-EV counts till they reached a plateau at around 1000-fold dilution. **Top right:** median Violet- side scatter intensity was plotted against the dilution factor. That showed a steady Violet- side scatter intensity across the dilution range. **Bottom plot:** median fluorescence intensity for FITC plotted against the dilution factor. That also showed a steady median fluorescence intensity across the dilution range. Hence the first dilution has been chosen (the most concentrated).

## Detergent-treated controls

To assess purity and confirm that the detected events are membrane bound vesicles rather than protein complexes or lipoproteins from plasma. A Lactadherin-FITC stained L-EV sample was treated with 0.5% (in 0.1 µm-filtered PBS) of the non-ionic detergent Triton X-100 filtered by a 0.22 µm-filter (Triton X-100, Sigma-Aldrich, St Louis, MO, US) left for 1 minute at room temperature. The stained L-EV sample before and after detergent treatment and the positive events were compared in both. The events in the FITC positive L-EV gate decreased 98% of the originally measured events, indicating L-EV lysis and that the measured events were truly L-EVs.

## Trigger channel(s) and threshold(s)*

Based on the buffer only and unstained sample controls (3.1), the detection was triggered on the 405 nm Violet laser, height parameter (405/10 bandpass filter) at a threshold of 1500 arbitrary units and a V-SSC gain of 100, The buffer alone control (3.1) had an event rate of ~1000 events s−1.

## Flow rate/volumetric quantification*

Flow rate calibration was assessed using an integral instrument flow rate calibration function in the Cytexpert software, resulting in a flow rate of 10 µL/min. This was calibrated using weighed volumes of deionized water prior and after acquiring a known volume of the deionised water by the cytometer. The peristaltic pump and tubing were changed according to the manufacturers recommendations periodically to maintain accurate flow rate. And a histogram of time versus counts was always monitored during all runs to check stability of flow rate.

## Fluorescence calibration*

No fluorescence calibration has been done; all data were acquired using arbitrary flow cytometry units. Florescence stability and consistency was assessed befroe each run, eight peak rainbow beads (Spherotech, Illinois, USA) were run before each experiment to ensure that the position of each of the eight bead populations with increasing fluorescent intensities is consistent between runs. Also, “Gaintration” (gain optimization for each florescent channel) was performed using the Spherotech 8 peak rainbow beads together with “FCM pass” software “gaintration” function. This was done by triggering on Violet-SSC at the same threshold determined in component 4.1 which is the same used for all L-EV experiments. And the acquisition mode was set on high acquisition mode. Then a pseudo-colour plot with forward scatter height (FSC-H) on the X axis and side scatter height (SSC-H) on the Y axis was created and the singlet bead population was gated. The rainbow beads were acquired at the lowest flow rate of 10 µl/minute which is the same flow rate used for L-EV samples. At least 10,000 events were required for the rainbow beads gate. Then the rainbow beads were acquired at 12 different ascending gains starting at 100 arbitrary units for each detector and incremented till reaching 3000 arbitrary units. Then all the FCS files were exported and uploaded to FCMPASS software (v4.1.1, https://www.fcmpass.com/) were optimum gains to identify dim L-EV populations were selected based on the best separation of background noise from the dimmest rainbow bead population.

## Scatter calibration

Scatter calibration was performed using Rosetta (Exometry, The Netherlands), this was done by running the Rosetta beads which are National Institute of Standards and Technology (NIST) traceable beads of sizes ranging from 70 nanometres up to 1 µm. The beads were run at the same Violet site scatter threshold and gain used in the actual L-EV experiments. Then the FCS file for Rosetta beads was exported and loaded in the Rosetta Calibration software (Exometry, The Netherlands). Then using the Mie theory (taking into account the beads refractive index and the optical configuration of the flow cytometer) and Exometry software the arbitrary Violet side scatter units were changed into standardised units by creating a new parameter: diameter in nanometres. This allows for reproducibility of results between flow cytometers and laboratories as well as accurate size gating of the EVs. Also, this allows for the determination of the limit of detection of the flow cytometer, which was determined to be 215 nanometres, meaning that the flow cytometer can detect EVs greater than or equal to 215 nanometres in diameter (4).

## L-EV diameter/surface area/volume approximation

Not assessed

## L-EV refractive index approximation

Not assessed, but for Mie theory calculation for scatter calibration an L-EV refractive index of 1.48 for the L-EV shell (plasma membrane), and a refractive index of 1.38 for the L-EV core (cytosol) was used(5).

## L-EV epitope number approximation

Not assessed

## Completion of MIFlowCyt checklist*

Yes

## Calibrated channel detection range

Around 210 nm (limit of detection) to 1000 nm of L-EV diameter (upper L-EV gate boundary set).

| 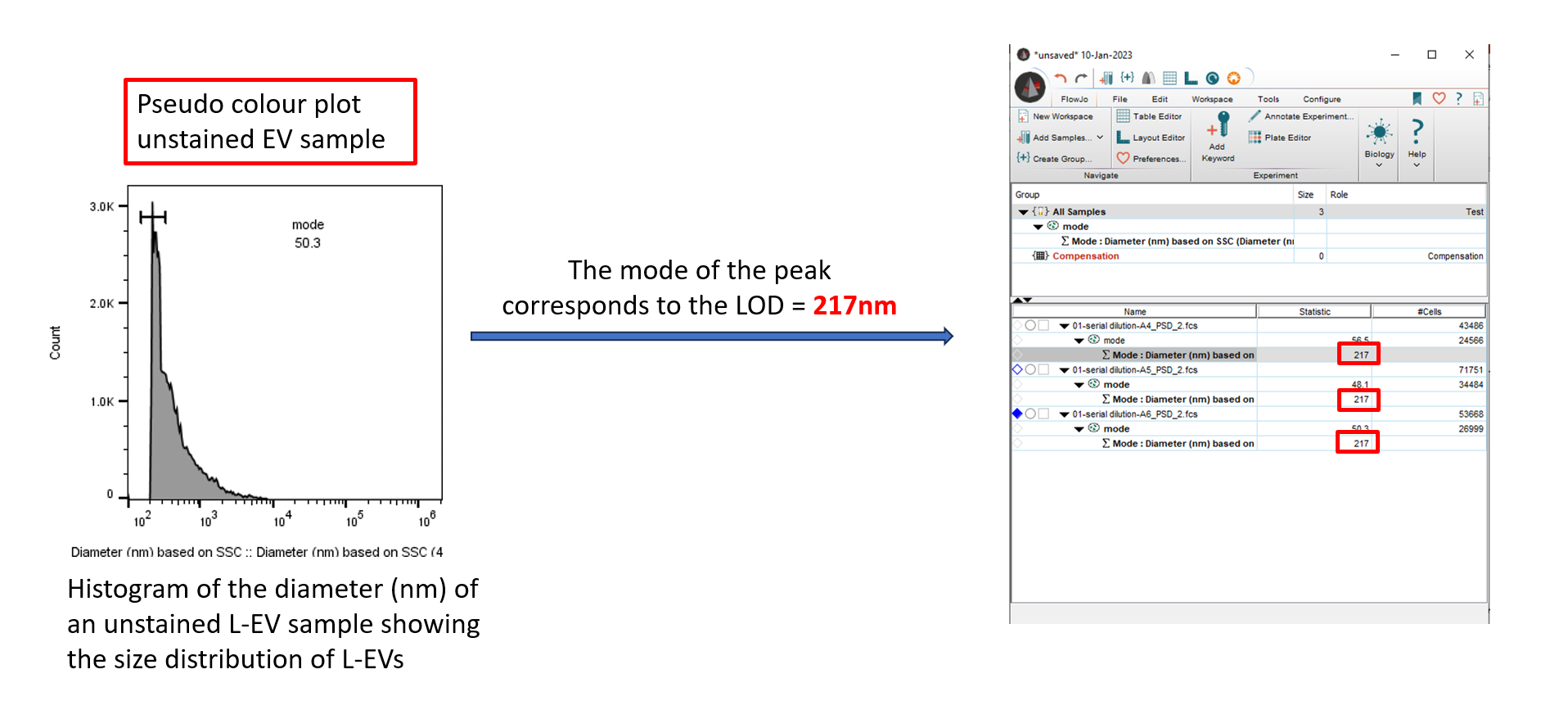  **Limit of detection. Left:** Histogram of the diameter of L-EVs in nm of an unstained L-EV sample showing the size distribution. **Right:** The mode of the peak was obtained on FlowJo and was considered as the lower limit of detection of the flow cytometer. |
| --- |

## L-EV number/concentration

L-EVs were counted by adding 20 µL of PE- florescent, small sized (3 µm) counting beads of known concentration (MP count beads, 7804, Biocytex, Marseille, France) to each stained sample just prior to analysis on the FCM. The beads where vortexed for 10 seconds just before use to re-suspend the beads evenly in solution. After the MVs staining was terminated, 20 µL of MP-Count Beads was spiked into each well containing the stained sample. The beads were acquired with each sample by the FCM, then a PE-H x SSC-H cytogram was created and then an ‘MP-Count’ gate was set around the dense cloud of the beads avoiding the doublets (as shown in Figure S2). Finally, to calculate the counts of L-EVs/ml in the samples the following formula was used (adapted from Brogan et al. (6))

L-EVs/μL = (***42,400* / no. of beads counted) x*** ***(no. of L-EVs counted per well) x dilution factor)***

***Number of mls of plasma (0.1 ml)***

*Number of MP-Count Beads placed in each well

## L-EV brightness

Not assessed

## Sharing of data to a public repository

FCS files and the analysis workspace will be uploaded to FlowRepository when this project is finished.

## Gating strategy for L-EVs by flow cytometry

To establish a size gate for L-EVs, an upper limit was set at 1000 nm of L-EV diameter (calibrated violet side scatter parameter). The gate for lactadherin-FITC was set above the background autofluorescence of an unstained sample and FMO controls.

# Mass spectrometry methods

## Sample Preparation

Concentrated L-EVs were resuspended in 40 µl of 8M Urea/50Mm tris hydrochloride. The protein samples were reduced by adding 8mM DTT, and mixing (thermomixer 1100rpm, 30°C) for 60 min and carboxylated by adding 20 mM iodoacetamide and mixing (thermomixer 1100rpm, 30°C) for 30 min in the dark. The samples were then diluted with 50 mM Tris HCL to bring the urea concentration below 2M. It is important that the Urea concentration is brought below 2M to prevent inhibition of trypsin. Each sample was digested overnight with sequencing grade trypsin by adding 1ug of trypsin to each sample (Promega, V5111). Digestion was terminated by adding formic acid to 1% final concentration (7).

## Mass Spectrometry

Following trypsin digestion, C18 HyperSep SpinTips (Thermo Scientific) were used to clean the samples from interfering molecules such as the salts and detergents used in peptide preparation. Each sample was analysed in duplicate on a Bruker timsTof Pro mass spectrometer connected to an Evosep One liquid chromatography system. Tryptic peptides from each sample were resuspended in 0.1% formic acid and loaded on to an Evosep tip. The autosampler is configured to pick up each tip, elute and separate the peptides using a set chromatography method, in this case 30 samples a day. Each sample was eluted from its Evotip onto a 15 cm, 150 µm internal diameter. analytical column packed with 1.5 µm C18 AQ reverse phase media (EV-1137 Performance Column – 30 samples/day, Evosep). Peptides were delivered to the analytical column in buffer a (LC/MS grade water/0.1% formic acid) and were separated with an increasing buffer B gradient (LC/MS grade acetonitrile/0.1% formic acid) over 44 minutes a flow rate of 0.5 µl/min (8). The mass spectrometer was operated in positive ion mode, at a capillary voltage of 1400 V, dry gas flow of 3 L/min and a dry temperature of 180°C. The instrument was operated in trapped ion mobility spectrometry (TIMS) mode during acquisition of all data. Trapped ions were selected for MS/MS using parallel accumulation serial fragmentation (PASEF). A scan range of (100-1700 m/z) was performed at a rate of 5 PASEF MS/MS frames to 1 MS scan with a cycle time of 1.03 seconds (9).

## Fragpipe Analysis (affinity purification-mass spectrometry/proteomics/phospho-proteomics)

The raw mass spectrometry data was searched against the Homo sapiens proteome on Uniprot Swissprot database (reviewed) using the Fragpipe proteomics pipeline (version 20.0) with specific parameters for trapped ion mobility spectra data dependent acquisition (TIMS DDA). For label free quantitation analysis, the LFQ-MBR workflow was selected. MS Fragger parameters include: percursor mass tolerance +/-20ppm, fragment mass tolerance +/-20ppm, strict trypsin, fixed modification; carbamidomethylation of cysteine, variable modifications; oxidation of methionine and N-terminal acetylation (10).

# Supplementary Figures and Tables


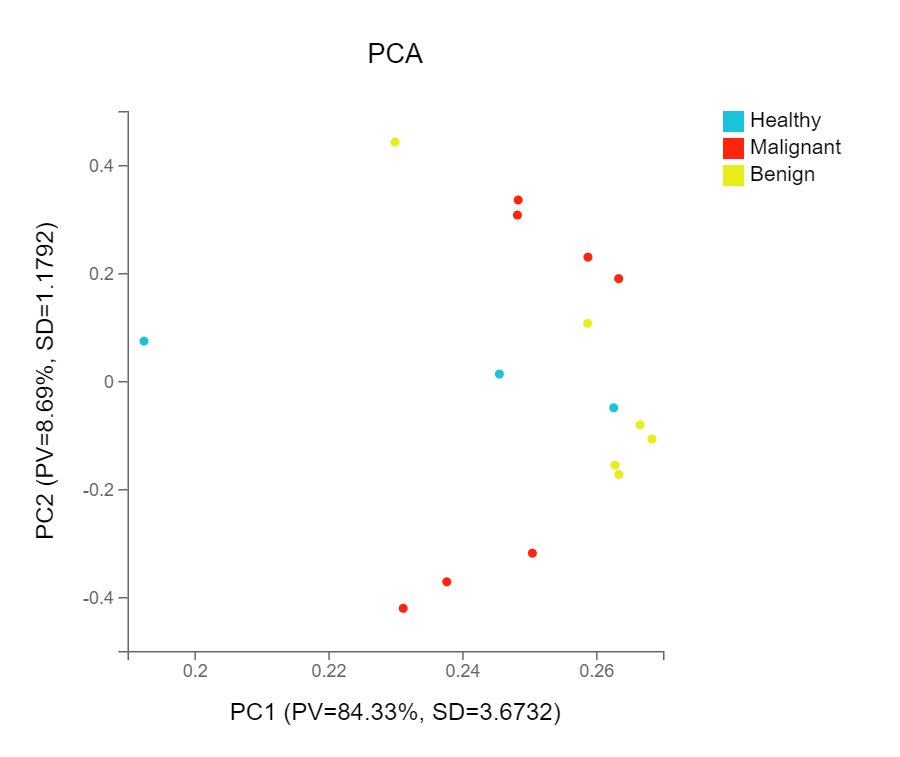


Figure S1: Principal component analysis plot of the miRNA sequencing data showing the interspersed clusitering of the samples. This has been generated on Dr Tom data analysis portal (BGI, Hong Kong).


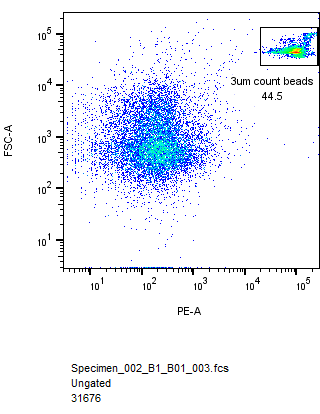


Figure S2: Pseudocolour plot showing PE-fluorescent 3 µm counting beads (MP count beads, Biocytex, Marseille, France) spiked into a stained MV sample (stained with Lactadherin-FITC) a rectangular gate is set around the main cloud of the bead population excluding the doublets according to manufacturer’s instructions. The event count obtained from these gated beads population from each well is used in the equation to calculate the large extracellular vesicles counts in each well per millileter of platelet poor plasma.

Figure S3: Quality control of large extracellular vesicles miRNA samples for next generation sequencing. RNA electropherogram shows a peak at around 25 neucleotides which is the miRNA size range. Small RNA quality and concentrations were analysed on an Agilent Bioanalyzer 2100 (Agilent technologies, USA). This representative sample had an RNA concentration of 0.357 ng/µl, RNA integrity number of 1.7, and 28S/18S ribosomal RNA ratio of zero reflecting absence of ribosomal RNA contamination of L-EV miRNA.


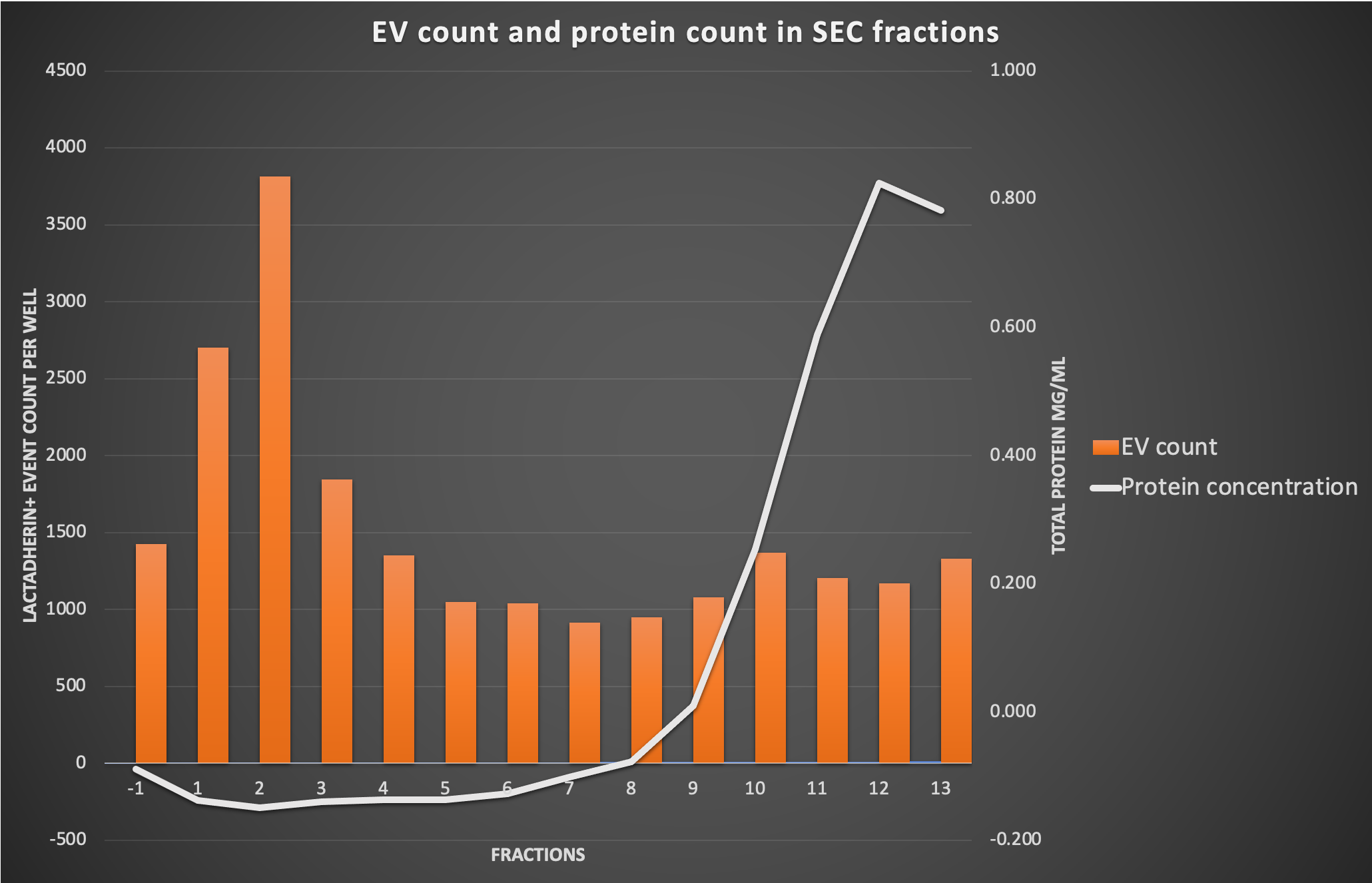


Figure S4: Large extracellular vesicle (L-EVs) concentrations in each fraction isolated from the plasma by a combination of differnetial centrifugation and size exclusion chromatography (SEC) meausred by flow cytometry and stained by lactadherin-FITC. Protein concentration of each fraction isolated by SEC from the plasma meausred by BCA. The protein concentrations spiked in later fractions (fractions 11 and 12) containing substantially more protein than previous fractions. This figure depicts a bar chart, where the columns represent L-EV counts and the line graph represents protien concentraions. The plot shows that most L-EVs are enriched in fractions 1-4, while fractions 11 and 12 mainly contained contaminating co-isolated plasma proteins(11).

**
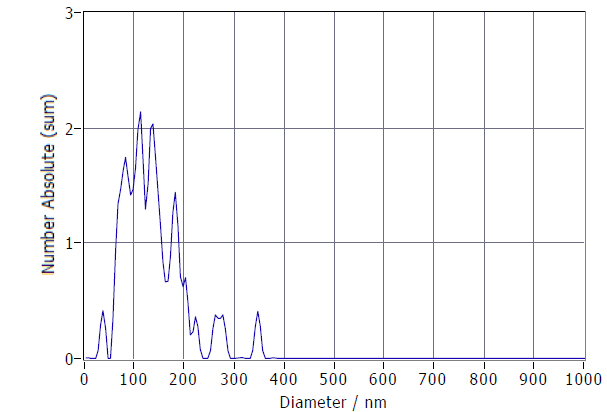
**

Figure S5: NTA analysis of L-L-EVdiameter distribution for a representative pooled and concnetrated SEC L-L-EVrich fractions 1–4. The median L-L-EVsize was 128nm, mode of 110nm. These measuremnts were made using ZetaView PMX-420 Quatt (Particle Metrix, Germany) equiped with a 488nm laser.


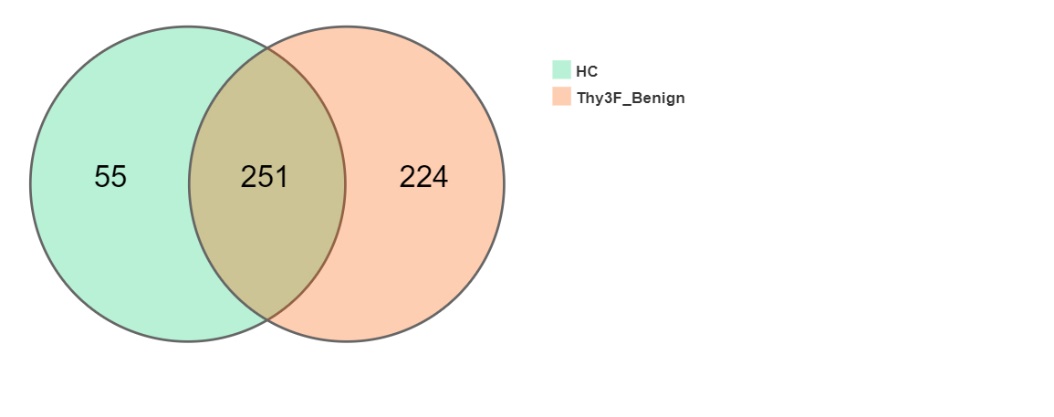

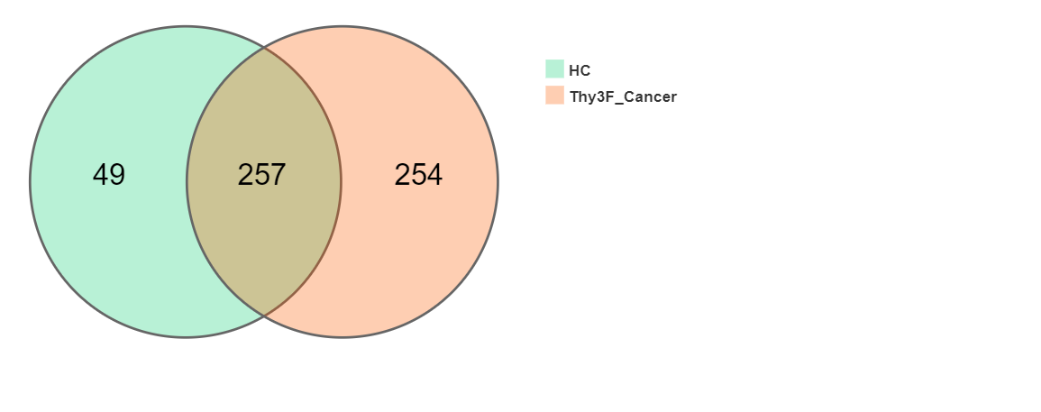


Figure S6: Venn diagram depicting the intersections between miRNAs identified in healthy controls (green), non-cancer (orange, left Venn diagram), and cancer (orange, right Venn diagram) Thy3f nodules, representing the number of miRNAs identified within the respective groups. HC: healthy controls.

**
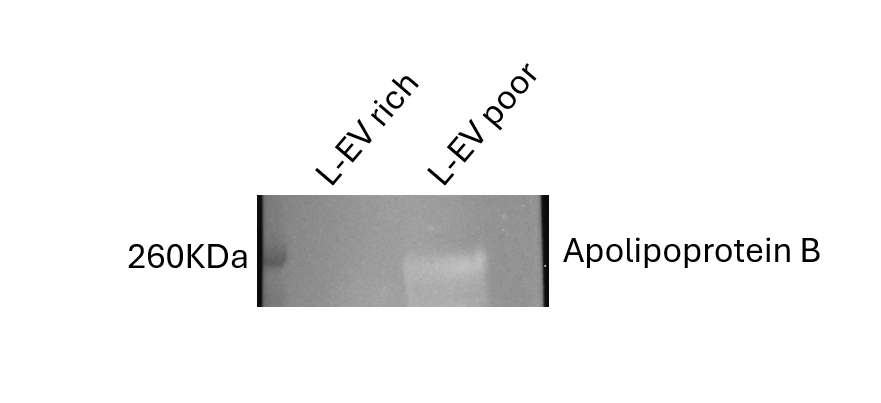
**

Figure S7: Representative western blot image depicting protien lysates of L-EV rich size exclusion chromatography (SEC) fractions (**left**) and L-EV poor SEC fractions (**right**) showing the lack of expression of Apolipoprotien B in the L-EV rich fractions denoting its purity and the absence of contaminating lipoproteins in the L-EV fractions. While Apolipoprotien B was only expressed in the L-EV poor fractions.

**
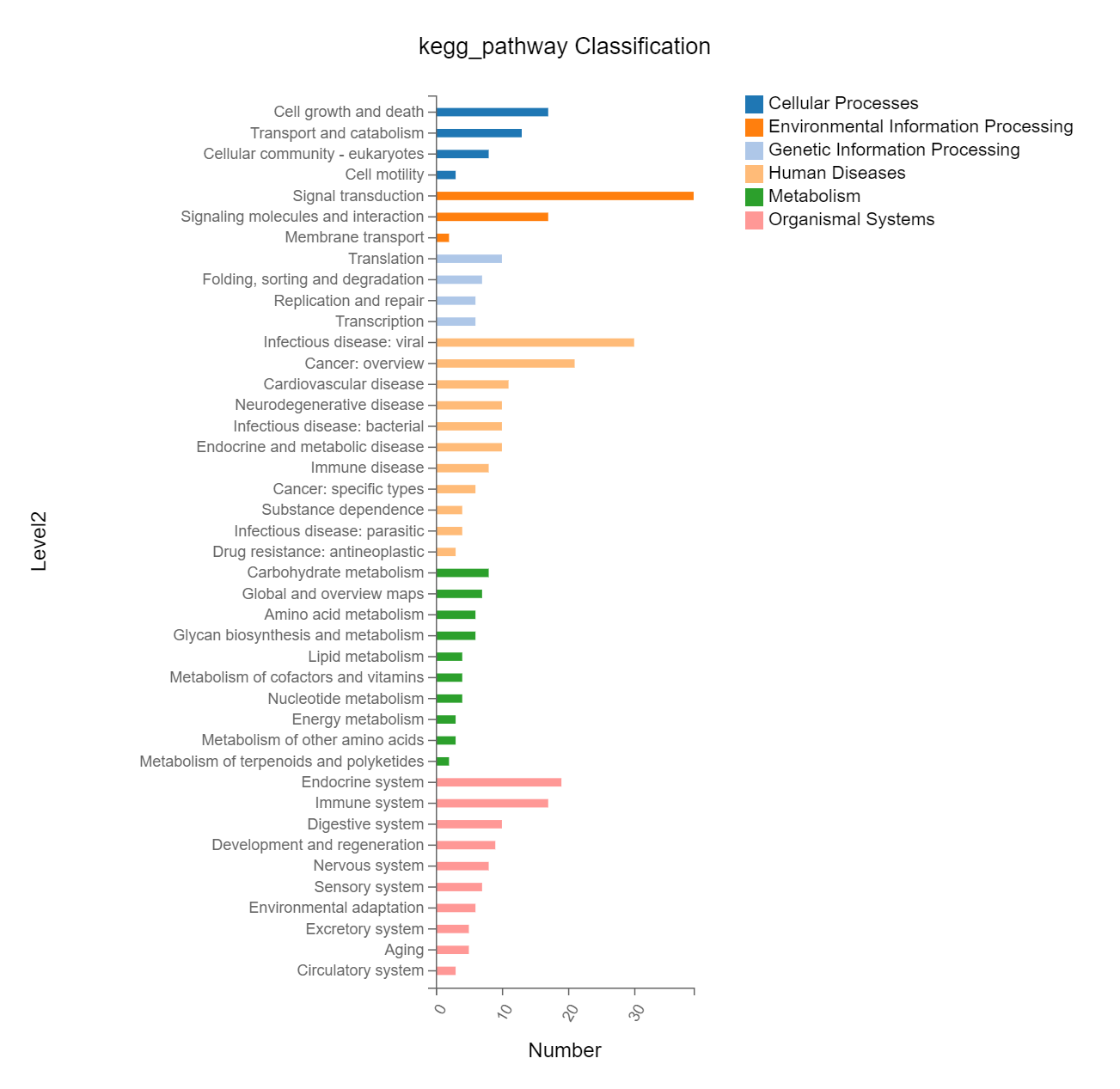
**

Figure S8: KEGG pathway analysis of the mRNA targets for hsa-miR-195-3p. mRNA targets were included in the analysis only when identified by three bioinforamtics target prediction databases: miRanda, TargetScan, and RNAhybrid

**
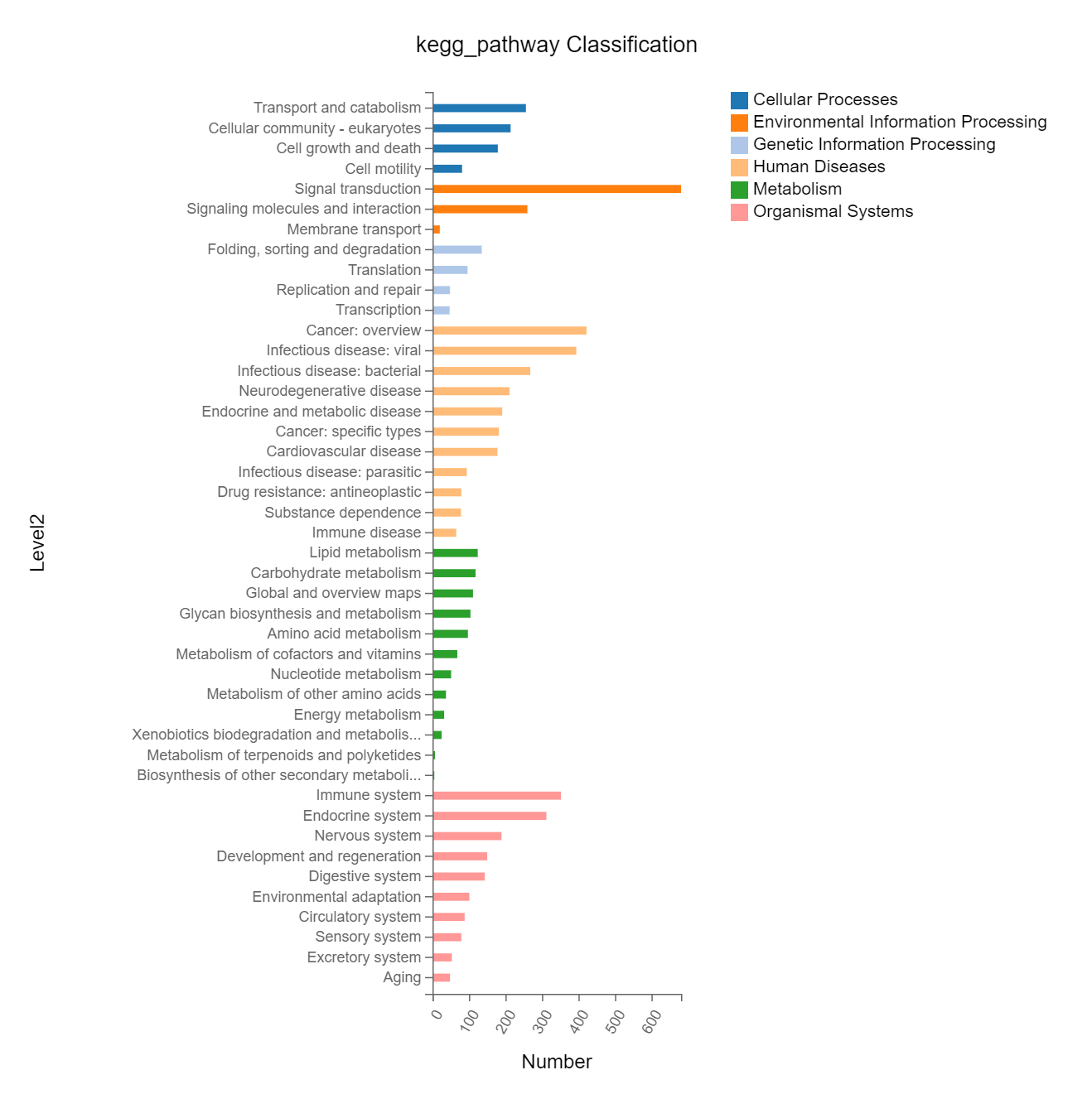
**

Figure S9: KEGG pathway analysis of the mRNA targets for hsa-mir-3176. mRNA targets were included in the analysis only when identified by three bioinforamtics target prediction databases: miRanda, TargetScan, and RNAhybrid

**
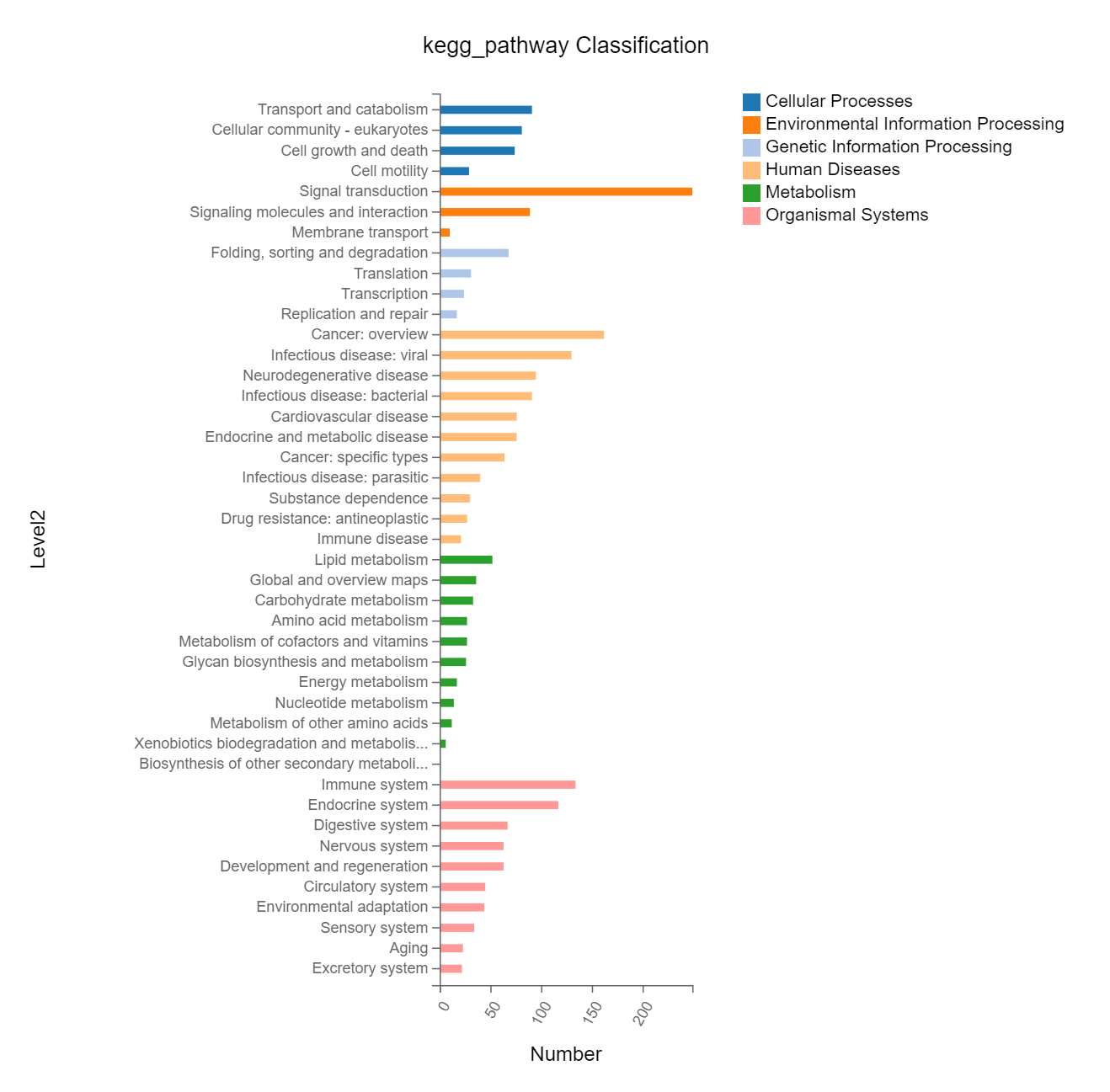
**

Figure S10: KEGG pathway analysis of the mRNA targets for hsa- mir-205-5p. mRNA targets were included in the analysis only when identified by three bioinforamtics target prediction databases: miRanda, TargetScan, and RNAhybrid

**
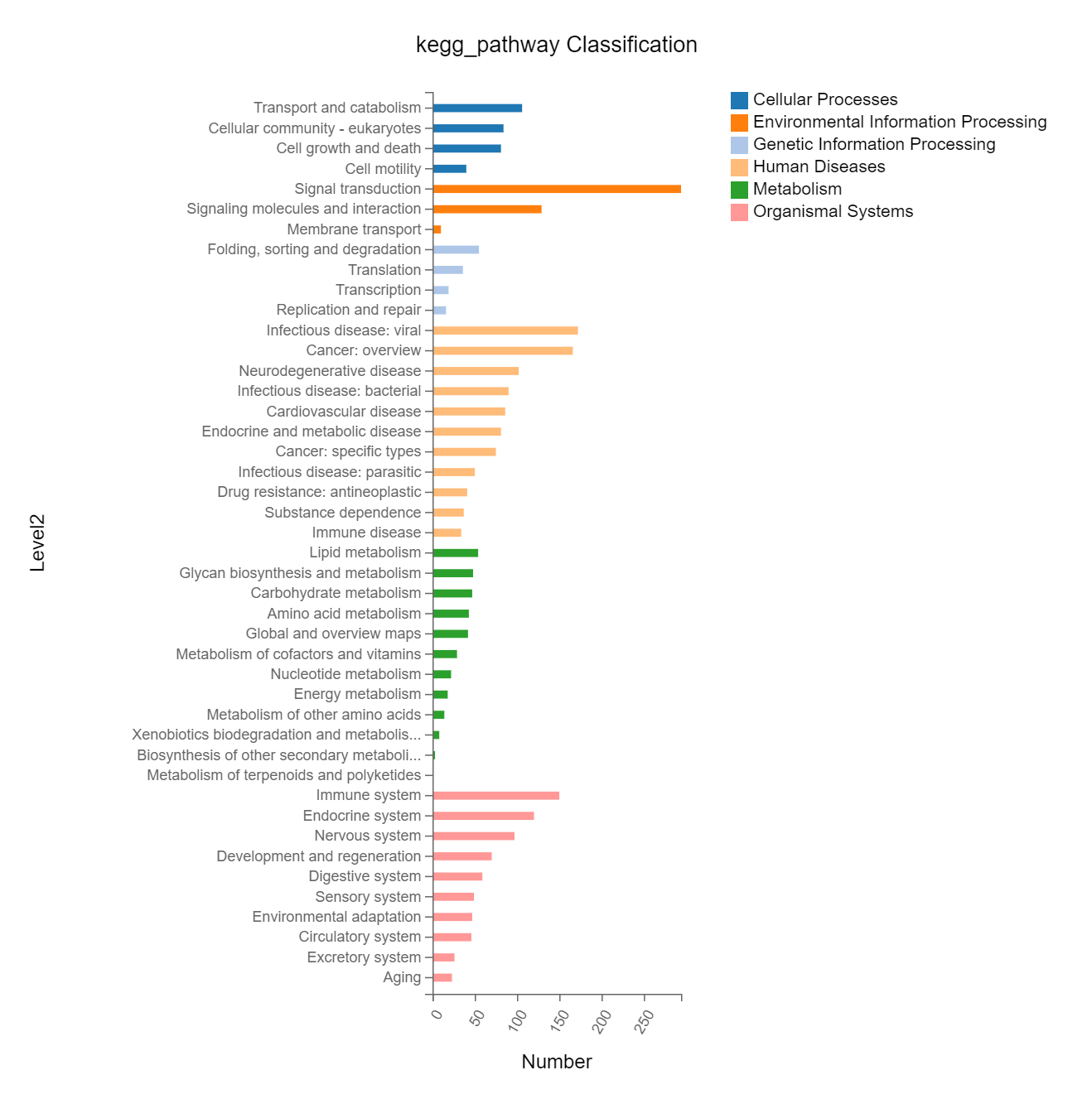
**

Figure S11: KEGG pathway analysis of the mRNA targets for novel-hsa-mir-208-3p. mRNA targets were included in the analysis only when identified by three bioinforamtics target prediction databases: miRanda, TargetScan, and RNAhybrid

**
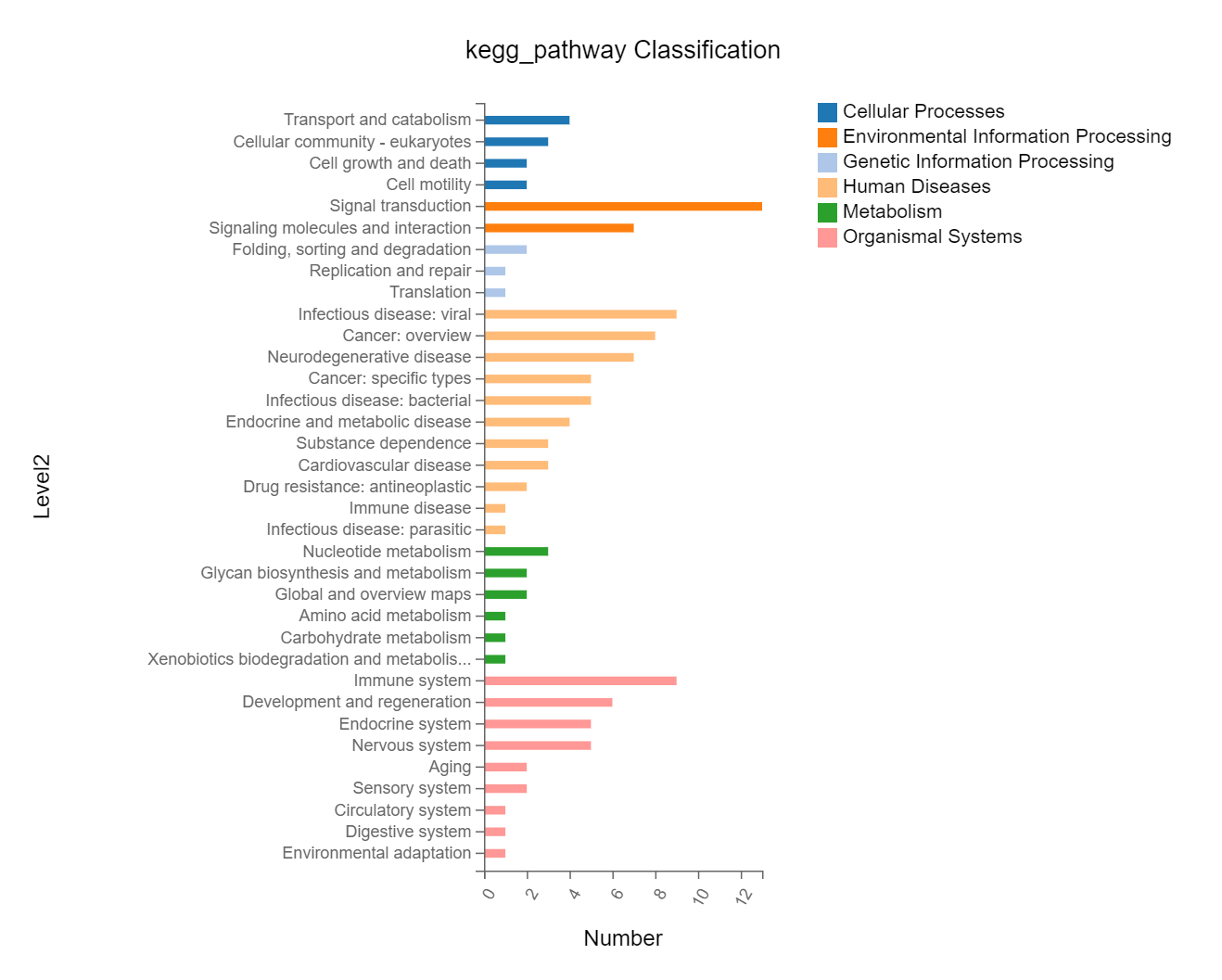
**

Figure S12: KEGG pathway analysis of the mRNA targets for hsa- mir-3529-3p. mRNA targets were included in the analysis only when identified by three bioinforamtics target prediction databases: miRanda, TargetScan, and RNAhybrid

**
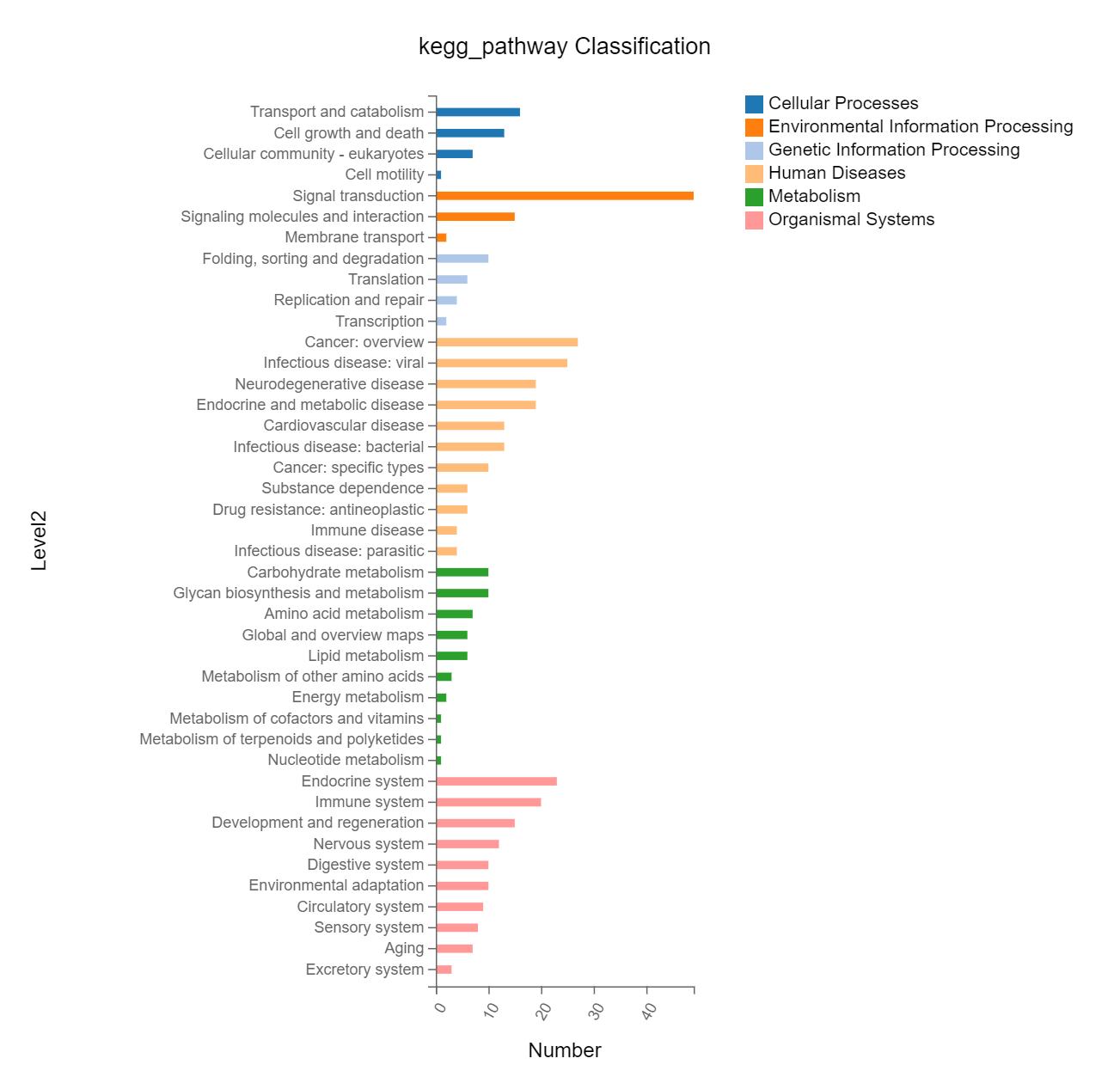
**

Figure S13: KEGG pathway analysis of the mRNA targets for hsa- let-7i-3p. mRNA targets were included in the analysis only when identified by three bioinforamtics target prediction databases: miRanda, TargetScan, and RNAhybrid

**Table S1: Details of all patients and healthy controls included in each analysis including demographics and thyroid nodules’ final diagnoses, nodule sizes, tumour stage for cancers**

| **Sample number** | **Final histopathological diagnosis** | **Tumour stage if applicable**  **Tumour size (mm)** | **Age** | **Gender** | **BMI** | **miRNA Sequencing** | **Proteomics** | **Flow cytometry** |
| --- | --- | --- | --- | --- | --- | --- | --- | --- |
| Cancer 1 | Mixed follicular and papillary architecture PTC (2 foci). infiltrative growth pattern, desmoplastic stroma, microscopic extrathyroidal extension.  Background: Focal chronic lymphocytic thyroiditis. | pT1a (m) N0  6.6mm and 2.4mm | 27 | F | 22.6 | + | - | + |
| Cancer 2 | FTC  Background: normal | pT1b NX M1 vertebral metastasis  18mm | 72 | F | 22.2 | + | - | + |
| Cancer 3 | Minimally invasive- oncocytic carcinoma, 3 foci capsular invasion, no vascular invasion and FV-PTC infiltrative growth pattern  Background thyroid: nodular disease | Oncocytic carcinoma: pT3a N0 Mx  54mm  FV-PTC: pT1a N0 Mx 0.7mm |  |  |  | + | - | + |
| Cancer 4 | FV-PTC encapsulated  Background: nodular hyperplasia | pT1a N0 Mx  3.5mm | 62 | F | 25.9 | + | - | + |
| Cancer 5 | FV-PTC unencapsulated Background: multinodular goitre | pT1a N0 Mx  2mm | 47 | F | 23.5 | + | + | + |
| Cancer 6 | FV-PTC, Desmoplastic stroma.  Background: nodular disease | pT1a N0 Mx  7mm | 74 | F | 36 | + | + | + |
| Cancer 7 | FV-PTC (2 foci) Unencapsulated.  Background thyroid: Multinodular hyperplasia | pT1a(m) pN0 Mx  4mm, 12mm | 40 | F | 27.9 | + | + | + |
| Cancer 8 | PTC papillary and follicular architecture, infiltrative growth pattern, multifocal (2 foci)  Background: mild lymphocytic thyroiditis | pT1a(m) Nx Mx  4mm each | 34 | F | 20.3 | + | + | + |
| Cancer 9 | FV-PTC multifocal (2 foci) (one with tall cell features), larger nodule: infiltrative growth pattern, desmoplastic stroma  Background: colloid nodules with oncocytic changes. | pT1b (m) N0 Mx  14mm and 1.2mm | 64 | F | 27.7 | + | - | + |
| Cancer 10 | Encapsulated FV-PTC (2 foci)  Background: focal lymphocytic thyroiditis | pT2 (m) N0 Mx  24.0 mm and 1.8 mm | 31 | F | 22.4 | + | + | + |
| Cancer 11 | FV-PTC: infiltrative growth pattern  Background: mild lymphocytic thyroiditis | pT3b N1b Mx  tumour: 19mm, lymph node: 15mm | 33 | F | 24.9 |  | + | + |
| Cancer 12 | FTC with 3 foci of lymphovascular and 3 foci of capsular invasion. PTC.  Background: moderate lymphocytic thyroiditis. | FTC: pT3a, N0, M0, 49mm  PTC: pT1a, 0.7mm | 54 | F | 27.9 | + | - | + |
| Cancer 13 | FTC with 3 foci of capsular invasion, 13 focus of lymphovascular invasion. Second tumour: unencapsulated FV-PTC.  Background: mild lymphocytic thyroiditis | FTC: pT2, N0  27mm  FV-PTC: pT1a, N0  4.2mm | 28 | F | 24 | + |  | + |
| Cancer 14 | WI-FTC oncocytic, with infiltrative, desmoplastic, LVI, and a FV-PTC  Background: normal | WI-FTC: pT3a N0 M1  56mm  FV-PTC: pT1a N0 1.2mm | 74 | M | 25 | + | + | + |
| Cancer 15 | WI-FTC with oncocytic areas and undifferentiated areas  Background: normal | pT3a N1 Mx  54mm | 61 | M | 34.7 | + | - | + |
| Cancer 16 | PTC mixed follicular and papillary architecture (2 foci), with Infiltrative growth pattern, desmoplastic stroma and lymphovascular invasion  Background: normal | pT2 (m) N1a M0  37mm and 17mm | 72 | F | 21.4 | - | + | + |
| Cancer 17 | PTC papillary and follicular architecture, with Several foci of Capsular invasion, Lymphovascular invasion.  Background thyroid: Scattered colloid nodules | pT2 N0 Mx  38mm | 50 | F | 29.9 | - | + | + |
| Cancer 18 | FV-PTC (2 foci), Infiltrative growth pattern  Background: nodular disease | pT1a (m) Nx Mx  2.1mm and 0.8mm | 60 | F | 41 | - | + | + |
| Cancer 19 | FV-PTC Encapsulated  Background: hyperplastic nodule | pT2, N0  40mm | 37 | F | 28.8 | - | + | + |
| Cancer 20 | FTC with 7 foci of capsular and 1 focus of lymphovascular invasion, Background: normal | pT3a, N0  54mm | 55 | M | 25.3 | - | + | + |
| Cancer 21 | MI-FTC Oncocytic  Background: normal | pT1b N0  14mm | 40 | F | 25 | - | + | + |
| Cancer 22 | MI-FTC Oncocytic  Background: normal | pT1b N0  13mm | 51 | F | 23.1 | - | + | + |
| Non-cancer 1 | NIFTP  Background: normal | -  30mm | 35 | F | 21.5 | + | - | + |
| Non-cancer 2 | Hyperplastic follicular nodule  Background: normal | -  16mm | 28 | F | 34.0 | + | - | + |
| Non-cancer 3 | Adenomatoid nodule  Background: normal | -  8mm | 43 | M | 22.9 | + | + | + |
| Non-cancer 4 | Follicular adenoma  Background: nodular disease | -  42mm | 60 | F | 38.5 | + | - | + |
| Non-cancer 5 | Adenomatoid nodule  Background: nodular disease | -  6mm | 34 | M | 22.6 | + | - | + |
| Non-cancer 6 | Adenomatoid nodule  Background: nodular disease | -  60mm | 44 | F | 30.3 | + | - | + |
| Non-cancer 7 | Adenomatoid nodule  Background: normal | -  28mm | 35 | F | 22.5 | + | + | + |
| Non-cancer 8 | Adenomatoid nodule  Background: mild nodular disease. | -  36mm | 54 | F | 21.1 | + | + | + |
| Non-cancer 9 | Follicular adenoma  Background: normal | -  30mm | 39 | F | 27.5 | + | - | + |
| Non-cancer 10 | Follicular adenoma  Background: normal | -  37mm | 66 | F | 23.9 | + | - | + |
| Non-cancer 11 | Follicular adenoma  Background: normal | -  19mm | 24 | M | 25.7 | + | + | + |
| Non-cancer 12 | Follicular adenoma with oncocytic change.  Background: normal | -  47mm | 62 | F | 31.1 | + | + | + |
| Non-cancer 13 | Oncocytic follicular adenoma  Background: normal | -  17mm | 36 | F | 31.6 | + | + | + |
| Non-cancer 14 | NIFTP  Background: normal | -  20mm | 26 | F | 21.3 | + | + | + |
| Non-cancer 15 | NIFTP  Background: normal | -  48mm | 30 | F | 19.3 | + | + | + |
| Non-cancer 16 | Follicular adenoma  Background: normal | 12mm | 38 | F | 26.5 | - | + | + |
| Non-cancer 17 | Follicular adenoma  Background thyroid: nodular hyperplasia | 38mm | 39 | F | 28.5 | - | + | + |
| Non-cancer 18 | Adenomatoid nodule  Background: normal | 54mm | 31 | M | 23.7 | - | + | + |
| Non-cancer 19 | Adenomatoid nodules  Background: nodular disease | 12mm and 20mm | 35 | F | 17.3 | - | + | + |
| Non-cancer 20 | NIFTP  Background: multinodular hyperplasia | 4mm | 72 | F | 27.6 | - | + | + |
| HC1 | - | - | 48 | F | 23 | + | + | + |
| HC2* | - | - | 31 | F | 21 | + | + | + |
| HC3* | - | - | 37 | M | 29 | + | + | + |
| HC4* | - | - | 37 | F | 25 | + | + | + |
| HC5* | - | - | 26 | M | 23 | + | + | + |
| HC6* | - | - | 31 | F | 22.5 | + | + | + |
| HC7* | - | - | 25 | F | 21 | + | + | + |
| HC8 | - | - | 70 | M | 29 | - | + | + |
| HC9 | - | - | 70 | F | 21 | - | + | + |
| HC10 | - | - | 61 | F | 22 | - | + | + |

*Pooling strategy for L-L-EVRNA obtained from Healthy control individuals for miRNA next generation sequencing is as follows: HC 2+3+4, and HC 5+6+7

Abbreviations: BMI: body mass index, PTC: papillary thyroid carcinoma, NIFTP: non-invasive follicular thyroid neoplasm with papillary like nuclear features, FV-PTC: follicular variant of PTC, FTC: follicular thyroid carcinoma, MI-FTC: minimally invasive FTC, WI-FTC: widely invasive FTC

**Table S2: Differentially expressed miRNAs between circulating L-EVs of** healthy controls, and patients with non-cancer, and cancer Thy3f nodules. The criteria for significant differential expression in DESeq2 was set at false discovery rates <0.05 and a fold change threshold, -1>log2FC> 1. All miRNA sequencing data was analysed on Dr Tom data analysis portal (BGI, Hong Kong).

|  | **MiRNA** | **Log2 Fold change** | **False discovery rate** |  | **MiRNA** | **Log2 Fold change** | **False discovery rate** |
| --- | --- | --- | --- | --- | --- | --- | --- |
| **Thy3f Cancer/ Healthy Controls** | | | | | | | |
| **Upregulated** | hsa-miR-103b | 6.99 | 0.04 | **Downregulated** | hsa-miR-1246 | -8.01 | 0.02 |
|  | hsa-miR-11400 | 8.88 | 0.01 |  | hsa-miR-1287-5p | -23.92 | 1.1e-10 |
|  | hsa-miR-128-3p | 6.52 | 0.009 |  | hsa-miR-129-5p | -23.27 | 2.7e-10 |
|  | hsa-miR-1301-3p | 21.81 | 0.00000002 |  | hsa-miR-149-5p | -4.18 | 0.01 |
|  | hsa-miR-181a-3p | 7.4 | 0.03 |  | hsa-miR-181d-5p | -7.59 | 0.02 |
|  | hsa-miR-20a-5p | 6.88 | 0.02 |  | hsa-miR-195-5p | -8.88 | 0.01 |
|  | hsa-miR-211-5p | 7.88 | 0.01 |  | hsa-miR-3200-5p | -25.77 | 2.0e-11 |
|  | hsa-miR-212-3p | 8.83 | 0.04 |  | hsa-miR-33a-5p | -7.19 | 0.03 |
|  | hsa-miR-376c-3p | 9.22 | 0.02 |  | hsa-miR-34b-3p | -10.50 | 0.01 |
|  | hsa-miR-423-5p | 10.34 | 0.01 |  | hsa-miR-3529-3p | -10.77 | 0.02 |
|  | hsa-miR-619-5p | 22.95 | 1.6e-10 |  | hsa-miR-361-5p | -7.30 | 7.19e-3 |
|  | hsa-miR-941 | 9.23 | 0.03 |  | hsa-miR-379-3p | -23.93 | 1.1e-10 |
|  | novel-hsa-miR189-5p | 6.69 | 0.02 |  | hsa-miR-4433a-3p | -24.54 | 7.5e-11 |
|  |  |  |  |  | hsa-miR-4725-3p | -23.98 | 1.1e-10 |
|  |  |  |  |  | hsa-miR-487a-3p | -24.23 | 8.8e-11 |
|  |  |  |  |  | hsa-miR-505-5p | -24.40 | 8.3e-11 |
|  |  |  |  |  | hsa-miR-518b | -9.69 | 0.02 |
|  |  |  |  |  | hsa-miR-548d-5p | -23.49 | 2.0e-10 |
|  |  |  |  |  | hsa-miR-5581-3p | -24.21 | 8.8e-11 |
|  |  |  |  |  | hsa-miR-671-3p | -23.72 | 1.6e-10 |
|  |  |  |  |  | hsa-miR-6803-3p | -23.37 | 2.5e-10 |
|  |  |  |  |  | hsa-miR-6884-5p | -23.32 | 2.5e-10 |
|  |  |  |  |  | hsa-miR-937-3p | -24.54 | 7.5e-11 |
|  |  |  |  |  | novel-hsa-miR119-3p | -24.56 | 7.5e-11 |
|  |  |  |  |  | novel-hsa-miR234-3p | -23.69 | 1.6e-10 |
| **Thy3f Benign/ Healthy Controls** | | | | | | | |
| **Upregulated** | hsa-miR-11400 | 9.73 | 0.01 | **Downregulated** | hsa-miR-1306-5p | -24.09 | 5.2e-13 |
|  | hsa-miR-128-3p | 6.99 | 9.16e-4 |  | hsa-miR-133a-3p | -8.24 | 3.20e-3 |
|  | hsa-miR-1301-3p | 8.71 | 0.03 |  | hsa-miR-149-5p | -5.47 | 5.02e-3 |
|  | hsa-miR-181a-3p | 6.90 | 0.04 |  | hsa-miR-206 | -6.67 | 0.02 |
|  | hsa-miR-205-5p | 21.34 | 4e-9 |  | hsa-miR-3200-5p | -25.61 | 6.4e-14 |
|  | hsa-miR-20a-5p | 7.02 | 0.05 |  | hsa-miR-361-3p | -23.22 | 2.3e-12 |
|  | hsa-miR-3176 | 22.01 | 5e-9 |  | hsa-miR-4725-3p | -24.79 | 2.3e-13 |
|  | hsa-miR-376c-3p | 8.05 | 5.37e-3 |  | hsa-miR-485-3p | -24.89 | 1.2e-15 |
|  | hsa-miR-423-5p | 23.02 | 1.2e-10 |  | hsa-miR-487a-3p | -24.10 | 5.2e-13 |
|  | hsa-miR-424-3p | 7.63 | 0.03 |  | hsa-miR-518b | -10.09 | 3.50e-3 |
|  | hsa-miR-4516 | 21.77 | 2e-9 |  | hsa-miR-548d-5p | -23.33 | 2.3e-12 |
|  | hsa-miR-486-3p | 8.85 | 3.41e-3 |  | hsa-miR-6513-3p | -23.29 | 2.3e-12 |
|  | hsa-miR-509-3p | 7.96 | 0.03 |  | hsa-miR-6803-3p | -23.20 | 3.1e-12 |
|  | hsa-miR-651-5p | 8.86 | 0.02 |  | hsa-miR-6884-5p | -23.03 | 3.6e-12 |
|  | novel-hsa-miR208-3p | 18.80 | 1.41e-6 |  | hsa-miR-937-3p | -24.43 | 3.2e-13 |
|  |  |  |  |  | novel-hsa-miR119-3p | -24.44 | 3.2e-13 |

Table (1): Demographics of patients with Thy3f nodules and healthy individuals, concentrations of total L-EVs and L-EV subpopulations expressing cancer markers: atypical chemokine receptor type 7 (CXCR7); extracellular matrix metalloproteinase inducer (CD147); and syndecan-4 (SDC4) and an epithelial marker epithelial cell adhesion molecule (EpCAM).

|  | **Age years, median**  **(Range)** | **Gender**  **Female (%)** | **BMI kg/m^2^, median (Range)** | **Nodule size (mm)**  **Median**  **(Range)** | **Total L-EV/ml (Interquartile range)** | **CXCR7 L-EV/ml (Interquartile range)** | **CD147 L-EV/ml (Interquartile range)** | **SDC4 L-EV/ml (Interquartile range)** | **EpCAM LEVL-EV/ml (Interquartile range)** |
| --- | --- | --- | --- | --- | --- | --- | --- | --- | --- |
| **Healthy controls**  **(n=16)** | 48  (25-75) | 20 (62.5%) | 24.3 (21.2-33) | - | 1.12x10^8^  (0.95-1.30x10^8^) | 1.01x10^8^  (0.86-1.16x10^8^) | 1.51x10^8^  (1.27-1.91x10^8^) | 1.36x10^8^  (1.20-1.80x10^8^) | 3.45x10^7^  (2.56-4.24x10^7^) |
| **Non-cancer Thy3f thyroid nodules**  **(n=14)** | 49.5  (24-73) | 6 (54.5%) | 24.97 (20.5-38.2) | 28mm  (4-60mm  ) | 1.99x10^8^  (1.61-3.21x10^8^) | 1.82x10^8^  (1.46-2.90x10^8^) | 2.30x10^8^  (1.83-2.76x10^8^) | 2.16x10^8^  (1.74-2.56x10^8^) | 5.57x10^7^  (4.40-6.69x10^7^) |
| **Cancer Thy3f thyroid nodules**  **(n=14)** | 46.3  (28-72) | 7 (70%) | 25.3 (23.1-28) | 19mm  (2-56mm  ) | 2.29x10^8^  (1.58-3.79x10^8^) | 2.09x10^8^  (1.44-2.74x10^8^) | 2.28x10^8^  (1.51-2.94x10^8^) | 2.37x10^8^  (1.64-2.95x10^8^) | 5.57x10^7^  (4.55-8.90x10^7^) |
| **p-value** | 0.2555 | 0.583 | 0.3252 | 0.389 | Thy3f cancer/ Thy3f non-cancer: >0.999  Thy3f cancer/ HC: 0.0001  Thy3f non-cancer/ HC: 0.0001 | Thy3f cancer/ Thy3f non-cancer: >0.999  Thy3f cancer/ HC: 0.0001  Thy3f non-cancer/ HC: 0.0001 | Thy3f cancer/ Thy3f non-cancer: >0.999  Thy3f cancer/ HC: 0.0376  Thy3f non-cancer/ HC: 0.0142 | Thy3f cancer/ Thy3f non-cancer: >0.999  Thy3f cancer/ HC: 0.0217  Thy3f non-cancer/ HC: 0.0193 | Thy3f cancer/ Thy3f non-cancer: >0.999  Thy3f cancer/ HC: 0.0007  Thy3f non-cancer/ HC: 0.0029 |

**References**

1. Vanderboom PM, Dasari S, Ruegsegger GN, Pataky MW, Lucien F, Heppelmann CJ, et al. A size-exclusion-based approach for purifying extracellular vesicles from human plasma. Cell Reports Methods. 2021;1(3):100055.

2. Gaspar LS, Santana MM, Henriques C, Pinto MM, Ribeiro-Rodrigues TM, Girão H, et al. Simple and Fast SEC-Based Protocol to Isolate Human Plasma-Derived Extracellular Vesicles for Transcriptional Research. Mol Ther Methods Clin Dev. 2020;18:723-37.

3. Buntsma NC, Shahsavari M, Gąsecka A, Nieuwland R, van Leeuwen TG, van der Pol E. Preventing swarm detection in extracellular vesicle flow cytometry: a clinically applicable procedure. Res Pract Thromb Haemost. 2023;7(4):100171.

4. Welsh JA, Van Der Pol E, Arkesteijn GJA, Bremer M, Brisson A, Coumans F, et al. MIFlowCyt-EV: a framework for standardized reporting of extracellular vesicle flow cytometry experiments. J Extracell Vesicles. 2020;9(1):1713526.

5. van der Pol E, van Leeuwen TG, Yan X. Misinterpretation of solid sphere equivalent refractive index measurements and smallest detectable diameters of extracellular vesicles by flow cytometry. Scientific Reports. 2021;11(1):24151.

6. Brogan PA, Shah V, Brachet C, Harnden A, Mant D, Klein N, Dillon MJ. Endothelial and platelet microparticles in vasculitis of the young. Arthritis Rheum. 2004;50(3):927-36.

7. Howard J, Wynne K, Moldenhauer E, Clarke P, Maguire C, Bollard S, et al. A comparative analysis of extracellular vesicles (EVs) from human and feline plasma. Sci Rep. 2022;12(1):10851.

8. Bache N, Geyer PE, Bekker-Jensen DB, Hoerning O, Falkenby L, Treit PV, et al. A Novel LC System Embeds Analytes in Pre-formed Gradients for Rapid, Ultra-robust Proteomics. Mol Cell Proteomics. 2018;17(11):2284-96.

9. Meier F, Brunner AD, Koch S, Koch H, Lubeck M, Krause M, et al. Online Parallel Accumulation-Serial Fragmentation (PASEF) with a Novel Trapped Ion Mobility Mass Spectrometer. Mol Cell Proteomics. 2018;17(12):2534-45.

10. Yu F, Haynes SE, Teo GC, Avtonomov DM, Polasky DA, Nesvizhskii AI. Fast Quantitative Analysis of timsTOF PASEF Data with MSFragger and IonQuant. Mol Cell Proteomics. 2020;19(9):1575-85.

11. Théry C, Witwer KW, Aikawa E, Alcaraz MJ, Anderson JD, Andriantsitohaina R, et al. Minimal information for studies of extracellular vesicles 2018 (MISEV2018): a position statement of the International Society for Extracellular Vesicles and update of the MISEV2014 guidelines. J Extracell Vesicles. 2018;7(1):1535750.
